# *Hagenia* from the early Miocene of Ethiopia: Evidence for possible niche evolution?

**DOI:** 10.1002/ece3.7408

**Published:** 2021-03-23

**Authors:** Friðgeir Grímsson, Silvia Ulrich, Mario Coiro, Shirley A. Graham, Bonnie F. Jacobs, Ellen D. Currano, Alexandros Xafis, Reinhard Zetter

**Affiliations:** ^1^ Department of Botany and Biodiversity Research University of Vienna Vienna Austria; ^2^ Department of Systematic and Evolutionary Botany University of Zurich Zurich Switzerland; ^3^ Missouri Botanical Garden St. Louis MO USA; ^4^ Roy M. Huffington Department of Earth Sciences Southern Methodist University Dallas TX USA; ^5^ Departments of Botany and Geology & Geophysics University of Wyoming Laramie WY USA; ^6^ Department of Paleontology University of Vienna Vienna Austria

**Keywords:** paleoecology, pollen morphology, pollen ultrastructure, Rosaceae, Sanguisorbeae, tropical forest

## Abstract

Fossil pollen believed to be related to extant *Hagenia abyssinica* were discovered in the early Miocene (21.73 Ma) Mush Valley paleoflora, Ethiopia, Africa. Both the fossil and extant pollen grains of *H. abyssinica* were examined with combined light microscopy, scanning electron microscopy, and transmission electron microscopy to compare the pollen and establish their relationships. Based on this, the fossil pollen grains were attributed to *Hagenia*. The presence of *Hagenia* in the fossil assemblage raises the questions if its habitat has changed over time, and if the plants are/were wind pollinated. To shed light on these questions, the morphology of extant anthers was also studied, revealing specialized hairs inside the anthers, believed to aid in insect pollination. Pollen and anther morphology are discussed in relation to the age and origin of the genus within a molecular dated phylogenetic framework, the establishment of complex topography in East Africa, other evidence regarding pollination modes, and the palynological record. The evidence presented herein, and compiled from the literature, suggests that *Hagenia* was an insect‐pollinated lowland rainforest element during the early Miocene of the Mush Valley. The current Afromontane habitat and ambophilous (insect and wind) pollination must have evolved in post‐mid‐Miocene times.

## Graphical abstract

This study shows that modern counterparts of fossil taxa might not be ideal to infer ecological or environmental traits, such as pollination, elevation, temperature, and precipitation on fossil plants or their assemblages. It is clear that since the origin of *Hagenia* its ecological requirements have changed significantly.

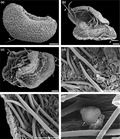

## 1 INTRODUCTION

Mountains are home to a substantial proportion of biological diversity, especially at tropical latitudes (Spehn et al., 2011). The origin of mountain biotas is rather complex, being driven by both geological and biological processes (Rahbek et al., 2019). Mountain lineages originate both from local lowland lineages through niche shift (evolution) and from preadapted lineages through long‐distance dispersal (Merckx et al., 2015). However, these two processes, which vary from region to region, are complicated further by uplift and isolation dependent upon geological evolution and past climate changes. Plant and animal species found today in East African Highlands forests often have close relatives in the forests of Central and West Africa or among the separated peaks of East Africa's mountains (Faden, 1983; Mairal et al., 2017; Measey & Tolley, 2011). Within East Africa, montane communities are a distinct region of biotic significance referred to as the Eastern Afromontane Biodiversity Hot Spot (Mittermeier et al., 2004), which adds significance to understanding its origins. The complexity of Africa's tropical upland biogeographic history is apparent from disjunct species having varying divergence times as calculated by phylogenetic studies (e.g., Couvreur et al., 2008; Mairal et al., 2017). Geologically, the ages of the East African Highlands vary from Eocene to Pleistocene (Faccenna et al., 2019; MacGregor, 2015; Tadesse et al., 2003), further complicating the historical biogeography of Africa's montane forest taxa. The most definitive way to document the biogeographic history of extant taxa found among the highlands of tropical Africa today is to identify them, or their ancestors, in the fossil record. Herein, we report the presence of the montane endemic genus *Hagenia* J.F. Gmel. in the early Miocene of Ethiopia.

*Hagenia* J.F. Gmel. is a tropical African Afromontane tree genus in the family Rosaceae Juss., subfamily Rosoideae (Juss.) Arn. (Ericksson et al., 2003; Potter et al., 2007), with a single recognized living species, *Hagenia abyssinica* (Bruce) J. F. Gmelin. Phylogenetically, *H. abyssinica* is a member of the tribe Sanguisorbeae DC., subtribe Agrimoniinae J. Presl, and its nearest relative is *Leucosidea sericea* Eckl. & Zeyh., a monotypic genus endemic to southern African uplands (Eriksson et al., 2003; Potter et al., 2007). *Hagenia abyssinica* is a long‐lived, dioecious evergreen tree up to 25 m tall (Lange et al., 1997) whose seeds are wind‐dispersed (e.g., Ayele et al., 2017; Negash, 2010). Trees are slender with a short trunk, massive branches, a large rounded crown, and ridged flaky bark. Conspicuous golden silky hairs cover the undersides of pinnately compound serrated leaves and densely encircle the nodes on young stem growth. The flowers are dioecious, female flowers forming impressive, cascading, thick panicles up to 60 cm long composed of hundreds of tiny tubular green‐pink to reddish flowers, each about 8 mm in diameter. A short floral tube terminates in conspicuous colored calyx and epicalyx lobes. Male inflorescences are slightly less dense, and the flowers are orange to brown or cream to white with 8–20 stamens (e.g., POWO, 2019; Simion, 2018; UTPD, 2014).

**FIGURE 1:**
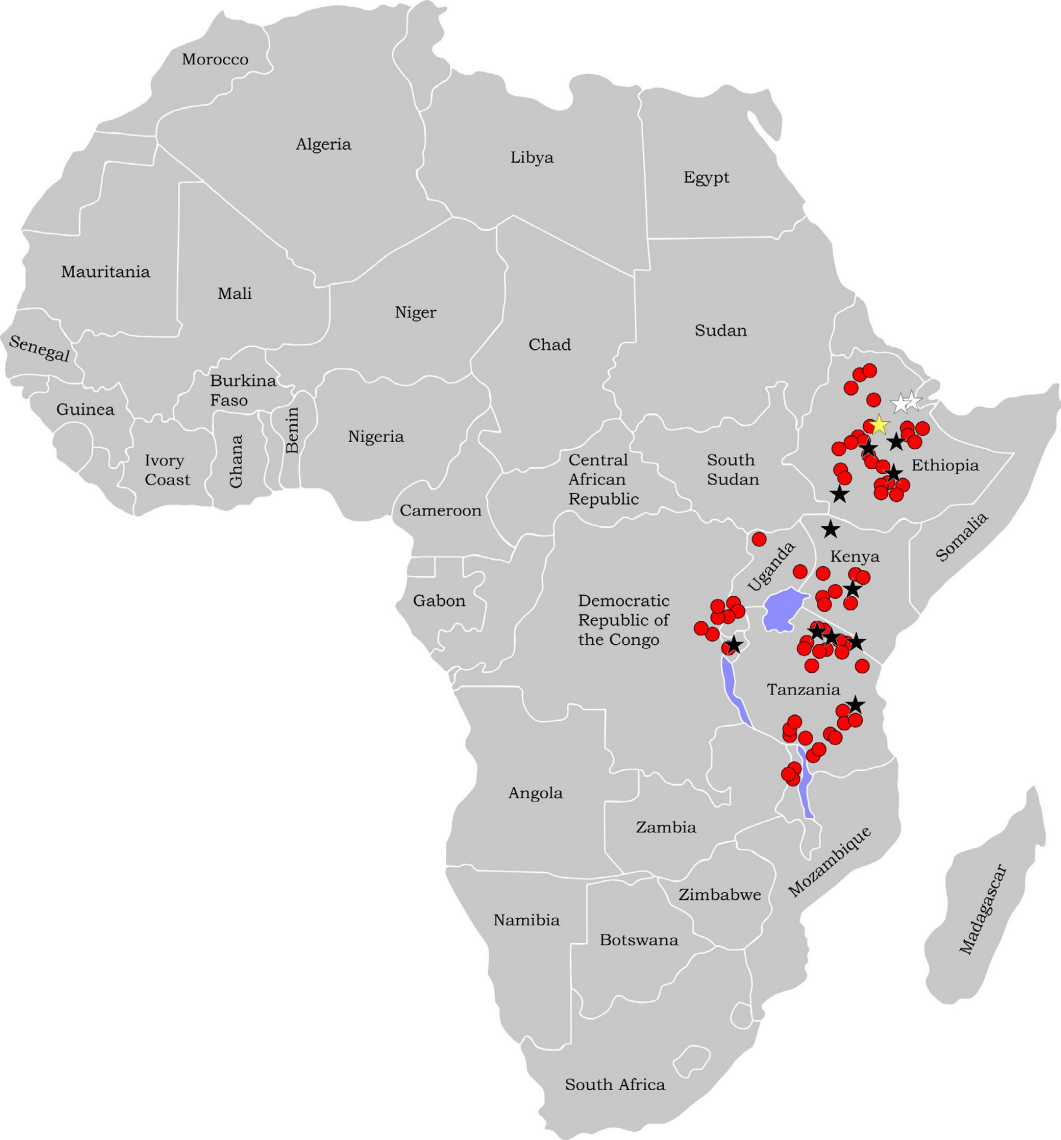
Distribution map of *Hagenia abyssinica* (red circles) based on GBIF Secretariat (2019). Location of the early Miocene (21.73 Ma) Mush Valley paleoflora, Ethiopia, is marked with a yellow star. Pliocene localities in Ethiopia are marked with white stars. Pleistocene localities in Ethiopia, Kenya, Tanzania, and Burundi are marked with black stars (for details see Discussion; The fossil record of *Hagenia*)

The current distribution of *H. abyssinica* includes Burundi, Democratic Republic of Congo, Eritrea, Ethiopia, Kenya, Malawi, Rwanda, Sudan, Tanzania, Uganda, and Zambia (Figure 1; e.g., UTPD, 2014; Simion, 2018; GBIF Secretariat, 2019; Habtemariam & Woldetsadik, 2019; POWO, 2019), primarily at upper elevations. As such, its natural environment is cool, wet montane forests in rich, moist soils. In eastern and central Africa, the genus occurs mainly from 2,000 to 3,000 m, with 1,000–1,500 mm of annual rainfall, but Hedberg (1989) and Friis (1992) report that it typically occurs between 2,300 and 3,400 m above sea level from northern Ethiopia and Sudan to southern Malawi and Zambia. The species, however, is known to tolerate much wider ecological conditions including drier forests and woodlands, and can be found at elevations from 1,800–4,300 m, under mean minimum and maximum temperatures from 5–36°C, and rainfall as low as about 400 mm a year (e.g., POWO, 2019; Simion, 2018; UTPD, 2014). It is also found on rocky soils or in disturbed areas such as roadsides and persists in grasslands formed following forest destruction. *Hagenia abyssinica* is thought to be a pioneer, light‐demanding species requiring higher soil temperatures to germinate (Fetene & Feleke, 2001; Lange et al., 1997; Young et al., 2017). *Hagenia abyssinica* often co‐occurs with *Hypericum revolutum* Vahl (the Hagenia‐Hypericum zone), *Schefflera volkensii* (Harms) Harms, *Juniperus procera* Hochst. ex Endl., and *Podocarpus falcatus* (Bussmann, 2006; Umer et al., 2007). In Ethiopia, some forests are dominated by *H. abyssinica* or uniquely dominated by a combination of *H. abyssinica* and *Juniperus procera* (African pencil‐cedar; AFT, 2009).

*Hagenia abyssinica* is recognized in Africa under a number of local names, kosso in Amharic, mlozilozi or mdobore in Swahili, and as African redwood in English (Orwa et al., 2009). It has become increasingly rare, endangered by the escalating destruction of forest lands due to human population growth. Many populations are scattered and disjunct throughout the range. Reduction in forests to open grassland is of concern with respect to *H. abyssinica* because it is an important traditional source of medicine for a variety of common ailments. It is still widely employed in the countryside as an antihelminthic against intestinal parasites such as tapeworms and other cestodes in both humans and animals (Assefa et al., 2010; Simion, 2018). In addition to antihelminthic properties, *H. abyssinica* has antimicrobial properties and is used to treat other gastrointestinal problems and a diverse array of diseases and complaints in both humans and animals. All parts of the plant are utilized, each designated for specific symptoms (e.g., Bekele & Reddy, 2014; Jima, 2018; Simion, 2018; Wolde et al., 2016a,b). In places where forest land has been cleared, one or more *H. abyssinica* trees can be seen in otherwise open fields, deliberately left for their value to the local community. This beautiful tree species is of widespread importance to the local population, not only for medicine but also for honey production, timber, dye, and in farming to control erosion and enhance soil fertility. Wood of the tree is used for construction, furniture making, dying of textiles, carving, and for firewood and charcoal (Simion, 2018).

The pollen morphology of *H. abyssinica* has been studied in relation to pollen of all other genera in tribe Sanguisorbeae, subtribes Agrimoniinae (incl. *Agrimonia* L., *Aremonia* Neck. ex Nestl., *Hagenia*, and *Leucosidea* Eckl. et Zeeyh.) and Sanguisorbinae (incl. *Cliffortia* L., *Acaena* L., *Margyricarpus* Ruiz et Pav., *Polylepis* Ruiz et Pav., *Poterium* L., *Poteridium* Spach, and *Sanguisorba* L.), by Pérez de Paz (2004) and Chung et al. (2010). These studies show that *H. abyssinica* pollen is unique within the tribe and easily identified using combined light microscopy (LM) and scanning electron microscopy (*SEM*). *Hagenia abyssinica* pollen has until now not been studied thoroughly with transmission electron microscopy (TEM). Nevertheless, the combined use of LM and *SEM* provides a uniquely diagnostic suite of characters that makes *Hagenia* the perfect pollen type to be discovered in paleopalynological samples. These would allow for documentation of the origin and evolution of the genus, together with its phytogeographic history. Unfortunately, fossil pollen grains of *Hagenia* are rarely reported from pre‐Pleistocene samples and thus far have not been documented using combined LM and *SEM*.

This study describes the morphology (LM, *SEM*) and ultrastructure (TEM) of extant and fossil *Hagenia* pollen from Africa. A comparison of fossil and extant characters provides the basis upon which evolutionary trends in the pollen morphology of the genus are discussed. Based on the paleoenvironmental reconstructions, conclusions are drawn regarding the paleoecology of *Hagenia* and how its ecological range may have shifted (niche evolution) since the early Miocene. The pollination of *Hagenia* is discussed in relation to palynological records, present day field observations from literature, morphology of anthers, and mode of anther dehiscence. Also, along with the *Hagenia* pollen, additional Sanguisorbeae (studied with combined LM and *SEM*) and Rosaceae fossils are used to calibrate divergence ages within the family/tribe and that of the African genus *Hagenia*.

## 2 MATERIAL AND METHODS

### 2.1 Origin and preparation of samples

#### 2.1.1 Pollen

Extant flower material (Table 1) from the Missouri Botanical Garden (MO) was prepared according to the protocol outlined in Grímsson et al. (2017), Grímsson et al. (2018) and Halbritter et al. (2018, pp. 103–105, Acetolysis the Fast Way). Fifty pollen grains from each sample were measured and studied with LM and *SEM*, and five pollen grains with TEM.

**TABLE 1:** Herbarium material used for this study

Taxon	Collector	Date	Coll. No.	Country	Elevation (m)	Herbarium
*Hagenia abyssinica* Willd.	J.J.F.E de Wilde	18.10.1969	5,836	Ethiopia	2,700	MO
*Hagenia abyssinica* Willd.	J. Lewalle	20.05.1979	5,739	Burundi	No record	MO
*Hagenia abyssinica* Willd.	Y.S. Abeid	30.07.2009	3,376	Tanzania	2,150	MO
*Hagenia abyssinica* Willd.	R.E. Yereau et al.	26.01.2008	7,008	Tanzania	1980	MO
*Hagenia abyssinica* Willd.	A.S. MKeya	26.08.1997	922	Tanzania	1,500	MO
*Hagenia abyssinica* Willd.	I.F. LaCroix	26.09.1987	4,822	Malawi	2,200	MO

Fossil *Hagenia* pollen was recovered from four samples within the lacustrine shales in the Mush Valley: MU7 at 4.0 m in the section of Currano et al. (2020, Figure 2), MU18 at 10.1 m, DDMU‐2 at 16.1 m, and DDMU‐1 at 17.7 m. The sedimentary rock samples were processed and fossil pollen grains extracted according to the method explained in Grímsson et al. (2008). The fossil pollen grains were investigated both by LM and *SEM* using the single grain method as described by Zetter (1989) and Halbritter et al. (2018, pp. 121–123). Five to ten fossil *Hagenia* pollen from each sedimentary sample were measured and studied with LM and *SEM*, and two to four grains with TEM. Additional fossil Sanguisorbeae pollen (see Appendix) originate from the early to middle Miocene of Assoyo de los Mineros, South America, and the middle Miocene of Botn, Iceland (Table 2). *SEM* stubs with fossil pollen produced under this study are stored in the collection of the Department of Paleontology, University of Vienna, Austria.

**FIGURE 2:**
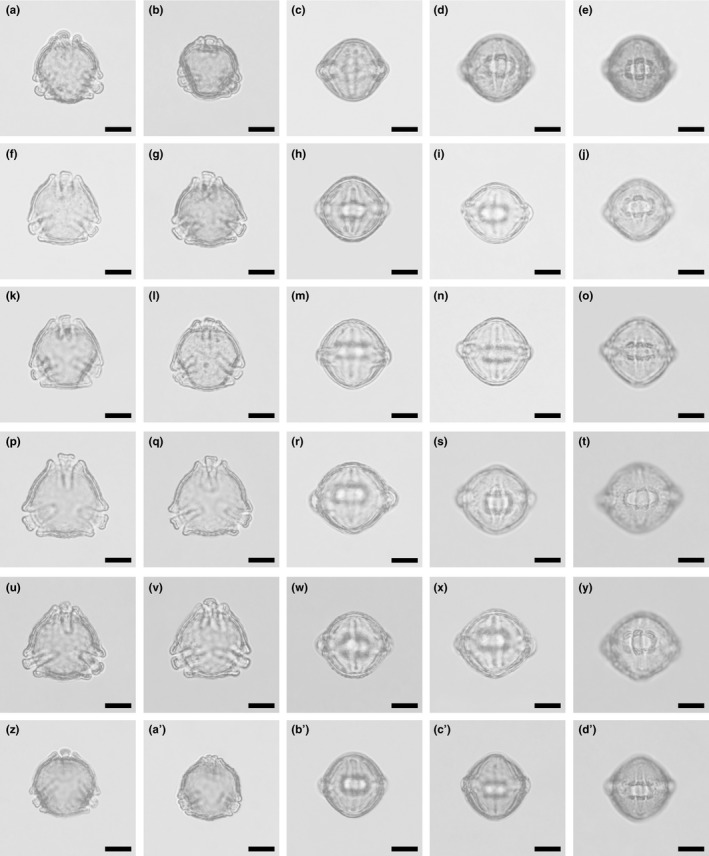
Light microscopy micrographs of *Hagenia abyssinica* from six different sites in Africa. (a–e) Ethiopia, 2,700 m a.s.l. (coll. J.J.F.E. de Wilde, 5,836 [MO]). (f–j) Burundi, elevation unknown, (coll. J. Lewalle, 5,739 [MO]). (k–o) Tanzania, 2,150 m a.s.l. (coll. Y.S. Abeid, 3,376 [MO]). (p–t) Tanzania, 1980 m a.s.l. (coll. R.E. Yereau et al., 7,008 [MO]). (u–y) Tanzania, 1,500 m a.s.l. (coll. A.S. MKeya, 922 [MO]). (z–d′) Malawi, 2,200 m a.s.l. (coll. I.F. LaCroix, 4,822 [MO]). (a, b, f, g, k, l, p, q, u, v, z, a′) Polar view, optical cross section. (c, h, i, m, n, r, w, x, b′, c′) Equatorial view, optical cross section. (d, e, j, o, s, t, y, d′) Equatorial view, high focus. Scale bars – 10 µm

**TABLE 2:** Information on sample sites

Locality	Arroyo de los Mineros	Mush	Botn
Taxon^a^	*Acaena* vel *Polylepis*	*Hagenia*	*Sanguisorba*
Location	Near the Arroyo de los Mineros (stream of miners), adjacent to Canadon Beta, north‐eastern Tierra del Fuego Province, coast of the Gulf of San Sebastian, Argentina, South America	Mush Valley, Debre Birhan Woreda, Ethiopia, Africa	Botn mine, Botnsdalur valley, Súgandafjörður fjord, Northwest Peninsula, Iceland, northern North Atlantic
Latitude and longitude (ca.)	52°42′S, 68°34′W	9°47΄N, 39°39΄E	66°04′N, 23°22′W
Lithostratigraphy	Cullen Formation	Unnamed lacustrine sediments	Selárdalur‐Botn Formation
Epoch/Age	Early to middle Miocene/Aquitanian to Serravallian	Early Miocene/Aquitanian	middle Miocene/Langhian
Numerical age (Ma)	21.3–11.1 (absolute dating)	22.63–21.73 (absolute dating)	ca 15 (absolute dating)
Age according to	Chrono‐, biostratigraphy	Chrono‐, lithostratigraphy	Chrono‐, magnetostratigraphy
For further info on the geological background, stratigraphy (S), paleoenvironment, paleoclimate, and plant fossils (P)	Vergel & Durango de Cabrera, 1988 (P), Durango de Cabrera & Vergel, 1989 (P); Zamaloa, 1996 (P), 1999 (S, P), 2000 (P), 2004 (S, P); Zamaloa & Romero, 1990 (P), 2005 (P); Zetter et al., 1999 (P); Garcia‐Massini et al., 2004 (P)	Danehy, 2010 (S/P); Pan et al., 2012 (S/P), 2014 (P); Bush et al., 2017(P); Tesfamichael et al., 2017 (S/P); Grímsson et al., 2019 (P); Currano et al., 2020 (S/P)	Moorbath et al., 1968 (S); Kristjánsson et al., 1975 (S), 2003 (S); McDougall et al., 1984 (S); Hardarson et al., 1997 (S); Grímsson et al., 2007a (S/P), 2007b (P), 2008 (P); Grímsson & Denk, 2007 (P); Grímsson & Símonarson, 2008a (P), 2008b (P); Denk et al., 2011 (S/P)

^a^ Earliest known fossil pollen records that include micrographs for verification.

For ultrastructural study of fossil and extant pollen, grains were prepared using the advanced TEM protocol by Ulrich and Grímsson (2020). Subsequent to *SEM* investigation, fossil pollen grains were transferred directly from *SEM* stubs, with a micromanipulator, into a final embedding mold filled with a mixture of Agar low‐viscosity resin (LV‐resin) and acetone for infiltration and final embedding. Pollen of *Hagenia abyssinica* (from Ethiopia, coll. J.J.F.E. de Wilde, 5,836 [MO]; from Burundi, coll. J. Lewalle, 5,739 [MO], from Tanzania, coll. Y.S. Abeid, 3,376 [MO], and from Malawi, coll. I.F. LaCroix, 4,822 [MO]) was first acetolyzed and then transferred directly with an acetone filled pipette into the final embedding mold. Following polymerization, ultrathin sections were made with a diamond knife on a Reichert Ultracut microtome and collected onto formvar film‐coated copper grids. For contrast, sections were stained with uranyl acetate (U, for 40 min), followed by lead citrate (Pb, for 3 min) (Hayat, 2000). For the detection of endexine, sections were treated with 1% aqueous potassium permanganate (KMnO_4_) solution for 5 min (Weber & Ulrich, 2010).

Technical note: The heat of the electron beam in TEM sometimes leads to problems when the pollen has been sputter coated and the formvar coated grids are thin. The formvar film adjacent to the gold cover can expand and rupture, resulting in a gap between the pollen wall and the gold cover, which can increase until the film completely ruptures (Ulrich & Grímsson, 2020).

#### 2.1.2 Anthers

Anthers from extant flowers (Table 1) were prepared for *SEM* to investigate the pollination mode of *H. abyssinica* by observing anther dehiscence and the presence/absence of pollen coatings and Ubisch bodies. The characteristic extent of dehiscence is well preserved in dried specimens (Castellanos et al., 2005). Open and closed anthers from different flowers were dissected, mounted on SEM stubs with double‐sided adhesive tape, and sputter coated with gold (Halbritter et al., 2018).

### 2.2 Phylogeny

The concatenated alignment and the consensus tree from Xiang et al. (2017) were downloaded from TreeBase (Piel et al., 2009) (matrix S22054). The single orthologous alignments were extracted using the python script split_concat_nexus.py (from https://gist.github.com/brantfaircloth/2999578), obtaining a total of 172 alignments. Taxa without any data were removed from the alignments using the program pxclsq from the phyx collection (Brown et al., 2017). Maximum Likelihood (ML) trees were generated for each ortholog group using RAxML ver. 8.0.0 (Stamatakis, 2014) run using the script raxml_wapper.pl (https://sco.h‐ its.org/exelixis/web/software/raxml/index.html). A GTR model with Gamma‐distributed rate variation and a proportion of invariable site model was employed for all tree searches. Five loci were then selected for the clock analysis using the approach of Smith et al. (2018) as implemented in the SortaDate script repository (Smith, 2019). First, the variance in the path between root and tips was calculated and then the proportion of splits present in the consensus tree of Xiang et al. (2017). Then, these metrics were combined and the five best loci were assessed. The five selected loci (ORTHOMCL46200, ORTHOMCL58800, ORTHOMCL97220, ORTHOMCL116510, ORTHOMCL122180) were then concatenated in a single alignment. The matrix was then trimmed to only include Rosaceae. Thirteen fossil taxa were added as tips, and the ages of these taxa were implemented as uniform distribution, taking into consideration the uncertainty in the dating of the strata where the fossils were recovered (see Tables 2 and 3). The placement of the fossil taxa was constrained using expert‐based assignation, while the backbone topology (of the extant taxa) was fixed to the consensus topology from Xiang et al. (2017). A dated analysis using the fossilized birth–death prior was run using MrBayes ver. 3.2.7 (Ronquist et al., 2012) as implemented on the CIPRES Science Gateway (Miller et al., 2010). The prior on the age of the tree was set as a uniform distribution between the oldest age for the oldest fossil in our analysis (56 Ma) and the age of the oldest eudicot fossil (125 Ma) (Coiro et al., 2019). A GTR plus gamma model was employed on five separate partitions for the five loci, with the substitution rates and the gamma shape parameters unlinked over the partitions. A single Independent Gamma Rate clock model was applied to all partitions. The sampling probability parameter of the fossilized birth–death prior was set to 0.02 (the proportion of extant species of Rosaceae sampled in our matrix), and the sampling strategy was set to ‘diversity’. Six independent metropolis coupled runs (with one cold chain and three heated chains) were run for 50,000,000 generations, sampling every 5,000 generations. Convergence of the mcmc chains was checked using Tracer, until the Effective Sample Size of most parameters reached a value higher than 200 or higher than 100. A consensus tree was summarized using the ‘allcompat’ options of the ‘sumt’ command.

**TABLE 3:** Fossil calibrations employed in our dated analysis

Fossil	Age	Calibration	Formation	Reference for age
*Amelanchier scudderi*	34.7–34	*Amelanchier*	Florissant Fm	Prothero and Sanchez (2004)
*Vauquelinia comptonifolia*	53.5–44.5	*Vauquelinia*	Green River Fm	Smith et al. (2003)
*Neviusia* sp.	52–48.7	*Neviusia*	Allenby Fm	Read (2000)
*Oemleria janhartfordae*	49.45–49.35	*Oemleria*	Tom Thumb Member, Klondike mountain Fm	Wolfe et al. (2003)
*Prunus wutuensis*	56.0–47.8	*Prunus* s.l.	Wutu Fm	Li et al. (2011)
*Holodiscus lisii*	34.7–34	*Holodiscus*	Florissant Fm	Prothero and Sanchez (2004)
*Spiraea* sp.	52–48.7	*Spiraea*	Allenby Fm	Read (2000)
*Fragaria* sp.	2.94–2.86	*Fragaria*	Lost Chicken sequence	Matthews et al. (2003)
*Rosa germerensis*	56–47.8	*Rosa*		Edelman (1975)
*Rubus acutiformis*	47.8–41.2	*Rubus*	Boscombe Sand Fm	British Geological Survey (2020)
*Cercocarpus myricaefolius*	34.7–34	*Cercocarpus*	Florissant Fm	Prothero and Sanchez (2004)
*Sanguisorba* sp.	15.97–13.82	*Sanguisorba*	Selárdalur‐Botn Fm	Table 2
*Hagenia* sp.	23.03–20.43	*Hagenia*	Unnamed lacustrine sediments	Table 2
*Acaena* vel *Polylepis*	15.97–13.82	*Acaena*, *Polylepis*	Cullen Fm	Table 2

## 3 RESULTS

### 3.1 Pollen descriptions

The pollen terminology follows Punt et al. (2007; LM) and Halbritter et al. (2018; *SEM*). Pollen of the single extant *Hagenia* species, *H. abyssinica*, is described first, followed by a description of the fossil. For practical reasons, the fossil pollen is classified as a morphotype (MT) named after the locality where the grains were found.

#### 3.1.1 *Hagenia abyssinica* (Bruce) J. F. Gmelin

##### Description

**FIGURE 3:**
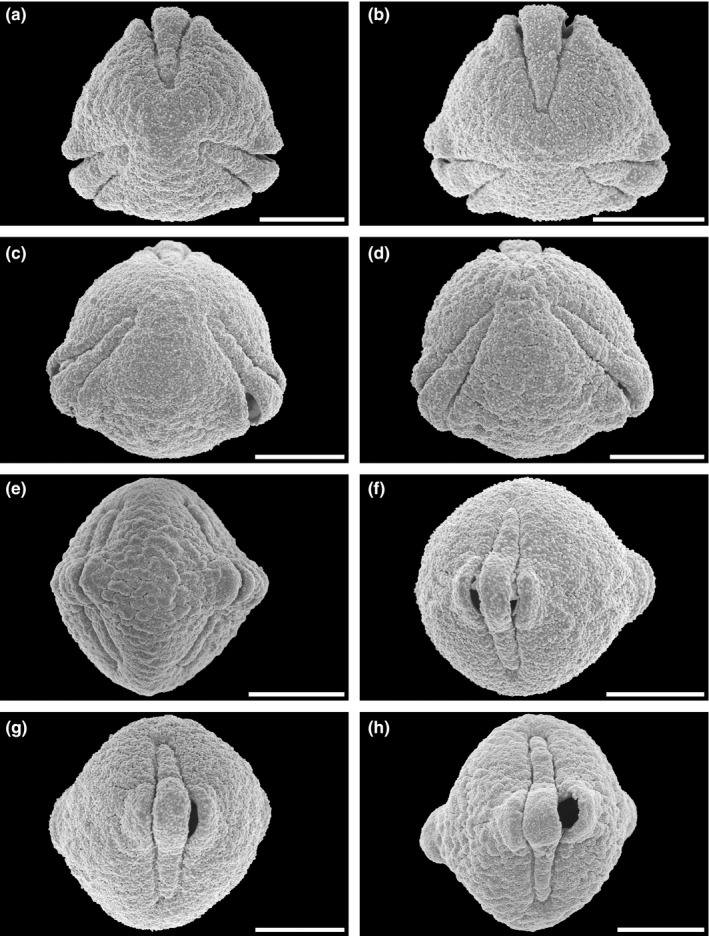
Scanning electron micrographs of *Hagenia abyssinica*. (a) Pollen in polar view (from Burundi, coll. J. Lewalle, 5,739 [MO]). (b) Pollen in polar view (from Ethiopia, coll. J.J.F.E de Wilde, 5,836 [MO]). (c) Pollen in oblique view (from Tanzania, coll. R.E. Yereau et al., 7,008 [MO]). (d) Pollen in equatorial view (from Tanzania, coll. A.S. MKeya, 922 [MO]). (e) Pollen in equatorial view (from Malawi, coll. I.F. LaCroix, 4,822 [MO]). (f) Pollen in equatorial view (from Ethiopia, coll. J.J.F.E. de Wilde, 5,836 [MO]). (g) Pollen in equatorial view (from Burundi, coll. J. Lewalle, 5,739 [MO]). (h) Pollen in equatorial view (from Tanzania, coll. Y.S. Abeid, 3,376 [MO]). Scale bars – 10 µm

**FIGURE 4:**
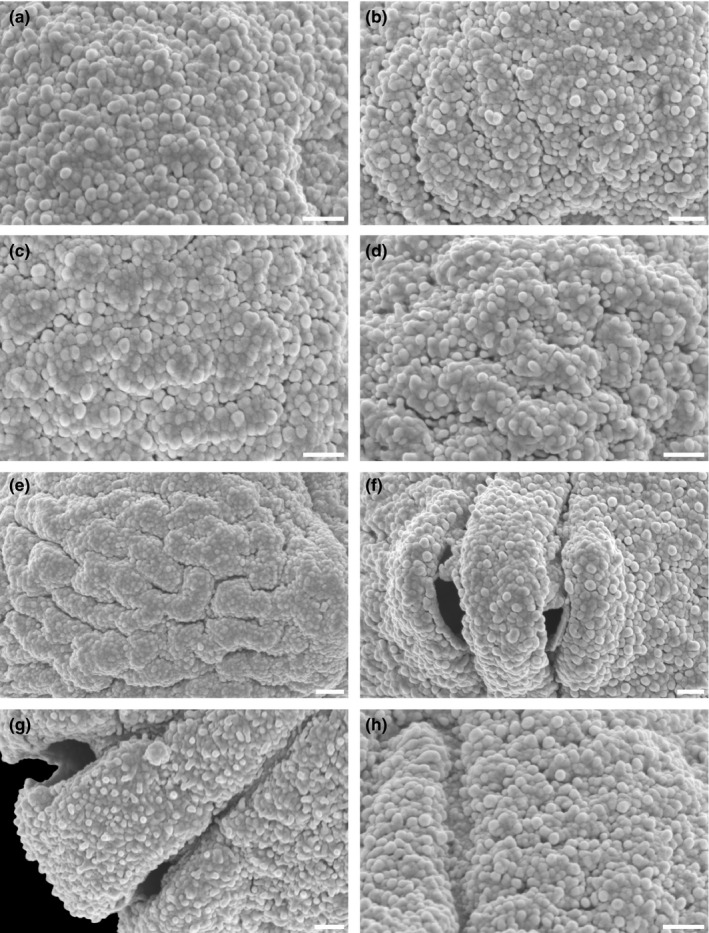
Scanning electron micrographs of *Hagenia abyssinica*. (a) Close‐up of polar area (from Tanzania, coll. R.E. Yereau et al., 7,008 [MO]). (b) Close‐up of intersection between polar area and interapertural area (from Burundi, coll. J. Lewalle, 5,739 [MO]). (c) Close‐up of interapertural area (from Tanzania, coll. Y.S. Abeid, 3,376 [MO]). (d) Close‐up of interapertural area (from Tanzania, coll. A.S. MKeya, 922 [MO]). (e) Close‐up of interapertural area (from Malawi, coll. I.F. LaCroix, 4,822 [MO]). (f) Close‐up of aperture, equatorial view, showing operculum (from Ethiopia, coll. J.J.F.E. de Wilde, 5,836 [MO]). (g) Close‐up of aperture, showing operculum, polar view (from Ethiopia, coll. J.J.F.E. de Wilde, 5,836 [MO]). (h) Close‐up of intersection between aperture and interapertural area (from Burundi, coll. J. Lewalle, 5,739 [MO]). Scale bars – 1 µm

**FIGURE 5:**
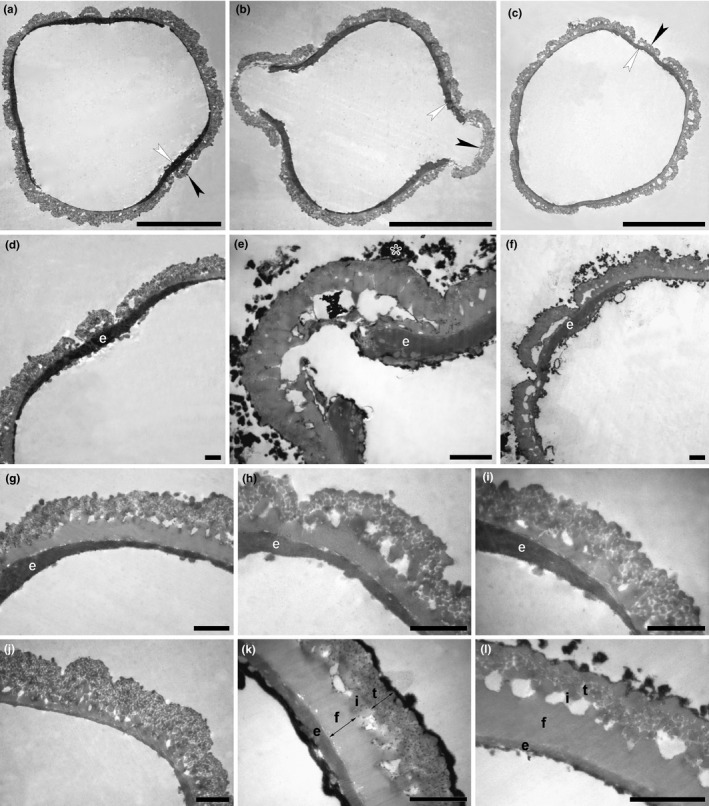
Transmission electron microscopy micrographs of *Hagenia abyssinica* from Africa. (a,b) Cross sections, polar view (a) and equatorial view (b), showing the operculate apertures (arrowheads), KMnO_4_ (from Tanzania, coll. Y.S. Abeid, 3,376 [MO]). (c) Cross section, oblique polar view, arrowheads indicating position of apertures, KMnO_4_ (from Malawi, coll. I.F. LaCroix, 4,822 [MO]). (d) Cross section, aperture area, showing thickening of endexine (e), polar view, KMnO_4_ (from Tanzania, coll. Y.S. Abeid, 3,376 [MO]). (e) Cross section, aperture area in equatorial view, remnants from the acetolysis mixture marked with asterisk, KMnO_4_ (from Ethiopia, coll. J.J.F.E. de Wilde, 5,836 [MO]). (f) Cross section, oblique polar view, KMnO_4_ (from Ethiopia, coll. J.J.F.E. de Wilde, 5,836 [MO]). (g–i) Cross sections, transition zone between aperture and interapertural area, showing thickening of endexine (e) toward aperture, KMnO_4_ (g, from Burundi, coll. J. Lewalle, 5,739 [MO]; (h) from Tanzania, coll. Y.S. Abeid, 3,376 [MO]; (i) from Malawi, coll. I.F. LaCroix, 4,822 [MO]. (j–l) Cross sections, interapertural area showing eutectate tectum (t, arrow), columellate infratectum (i), compact‐continuous foot layer (f, arrow), compact‐continuous endexine (e), KMnO_4_ (j), from Tanzania, coll. Y.S. Abeid, 3,376 [MO]; (k) from Burundi, coll. J. Lewalle, 5,739 [MO]; (l) from Ethiopia, coll. J.J.F.E. de Wilde, 5,836 [MO]). KMnO_4,_ potassium permanganate. Scale bars – 10 µm (a–c), 1 µm (d–l)

Pollen, monad, P/E ratio suboblate to subprolate, shape straight‐triangular to convex‐triangular dipyramid, straight‐triangular to convex‐triangular in polar view, elliptic to circular or rhombic in equatorial view; equatorial diameter 25–35 µm in LM, 22.7–27.7 µm in *SEM*, polar axis 21.3–30 µm in LM, 21–27 µm in *SEM*; tricolporate, apertures protruding, operculate, operculum 17.5–22.5 µm long in LM, 16.9–20.5 µm long in *SEM*, 2.5–6.3 µm wide in LM, 2.8–5.1 µm wide in *SEM*; exine 1.8–2.8 µm thick, nexine thinner than sexine (LM); tectate, columellate (*SEM*); sculpture psilate or rugulate in LM, nanogemmate, nanoclavate, and nanoechinate or rugulate with nanogemmate/clavate/echinate suprasculpture in *SEM* (Figures 2, 3, 4, 5; Tables 1 and 4).

**TABLE 4:** Pollen morphology and ultrastructure of extant and fossil *Hagenia*

	J.J.F.E de Wilde, 5,836 (MO)	J. Lewalle, 5,739 (MO)	Y.S. Abeid, 3,376 (MO)	R.E. Yereau et al., 7,008 (MO)
Equatorial diameter (LM)	25–30	27.5–30	27.5–30	31.3–35
Equatorial diameter (*SEM*)	24.1–25.6	24.6–25.7	25.1–26.9	25.1–27.2
Polar axis (LM)	21.3–27.5	22.5–25	26.3–27.5	27.5–30
Polar axis (*SEM*)	21–23	23.7–24.1	26–27	NO
Operculum lenght (LM)	18.8–21.3	17.5–21.3	20–22.5	20–22.5
Operculum lenght (*SEM*)	17–18	17.7–18.6	19–20	NO
Operculum diameter (LM)	3.8–5.0	3.8–5	3.8–5	3.8–6.3
Operculum diameter (*SEM*)	3.2–5.1	4.1–5.1	4.1–4.8	3.1–4.1
Sculpture interapertural area (*SEM*)	Rugulate	Rugulate	Rugulate	Rugulate
Suprasculpture (*SEM*)	Nanogemmate, clavate, echinate	Nanogemmate, clavate, echinate	Nanogemmate, clavate, echinate	Nanogemmate, clavate, echinate
Exine thickness (LM)	1.8–2.5	2.3–2.8	2–2.3	1.8–2.5
Ektexine thickness (TEM)	1.11	1.53	1.07–1.37	NO
Endexine, interaperturate (TEM)	0.11	0.2	0.14	NO
Anther hairs (*SEM*)	Present	Present	Present	Present
Ubisch bodies	Absent	Absent	Absent	Absent
Starch	Absent	Absent	Absent	Absent

	A.S. Mkeya, 922 (MO)	I.F. LaCroix, 4,822 (MO)	Total range extant	Mush (fossil, early Miocene)
Equatorial diameter (LM)	30–32.5	25–28.8	25–35	26.9–32.7
Equatorial diameter (*SEM*)	23.3–27.7	22.7–25.6	22.7–27.7	21.2–30.8
Polar axis (LM)	26.3–28.8	25–27.5	21.3–30	25–36.5
Polar axis (*SEM*)	23.3–24.6	22.7–23.7	21–27	24.8–31.9
Operculum length (LM)	20–22.5	20–22.5	17.5–22.5	17.3–25
Operculum length (*SEM*)	18.2–20.5	16.9–18.1	16.9–20.5	15.7–22.5
Operculum diameter (LM)	3.8–5.0	2.5–5	2.5–6.3	2.3–5
Operculum diameter (*SEM*)	2.8–4.4	3–4.2	2.8–5.1	2.2–5.1
Sculpture interapertural area (*SEM*)	Rugulate	Rugulate	Rugulate	Nanogemmate, clavate, echinate
Suprasculpture (*SEM*)	Nanogemmate, clavate, echinate	Nanogemmate, clavate, echinate	Nanogemmate, clavate, echinate	
Exine thickness (LM)	2.3–2.8	1.8–2.3	1.8–2.8	1.7–2.7
Ektexine thickness (TEM)	NO	0.92–1.38	0.92–1.53	1.0–1.07
Endexine, interaperturate (TEM)	NO	0.09	0.09–0.2	0.07
Anther hairs (*SEM*)	Present	Present	Present	NO
Ubisch bodies	Absent	Absent	Absent	NO
Starch	Absent	Absent	Absent	NO

Note:

All measurements are given in micrometers (µm). NO—not observed.

The ultrastructure (TEM) of the four samples studied is very similar. The variations in pollen wall thickness is due to different cutting angles. In acetolyzed pollen, the resistant exine is well preserved, whereas the intine and the living protoplast are absent. The pollen wall layers are clearly differentiated using potassium permanganate staining, as the endexine stains electron dense, producing a distinct contrast. The pollen wall constitutes a sporopollenin, thick structured tectate‐columellate ektexine, and a thin monolayered compact‐continuous endexine. The ektexine is between 0.9 and 1.53 µm thick, with thick continuous tectum (eutectate), columellate infratectum (with short columellae), and thick compact‐continuous foot layer. The tectum is granular throughout, composed of small homogeneous elements. The compact endexine forms a thin (0.09–0.2 µm) continuous layer, with increasing thickness in the aperture area. The operculum, formed by the ektexine, covers most of the aperture.

##### Remarks

*Hagenia abyssininca* pollen has previously been described and illustrated by Pérez De Paz (2004; LM and *SEM*) and Chung et al. (2010; *SEM*). The morphology presented by these authors is comparable to what is documented herein. The ultrastructure of *H. abyssinica* has not been studied successfully before, but see Jiang et al. (2019). The stratification of the pollen wall in *H. abyssinica*, especially the granular internal tectum, is a characteristic feature. This type of tectum also occurs in the closely related genus *Agrimonia* (Bombosi, 2016). A granular tectum is a rare feature in angiosperms but known for example in Asteraceae (Halbritter et al., 2018, p. 391). Within Rosaceae, the pollen wall stratification of *Hagenia* and *Agrimonia* is as far as known a unique feature in subtribe Agrimoniinae (tribe Sanguisorbeae), and further TEM studies are necessary for clarification.

#### 3.1.2 Mush MT, pollen close to *Hagenia abyssinica*

##### Description

**FIGURE 6:**
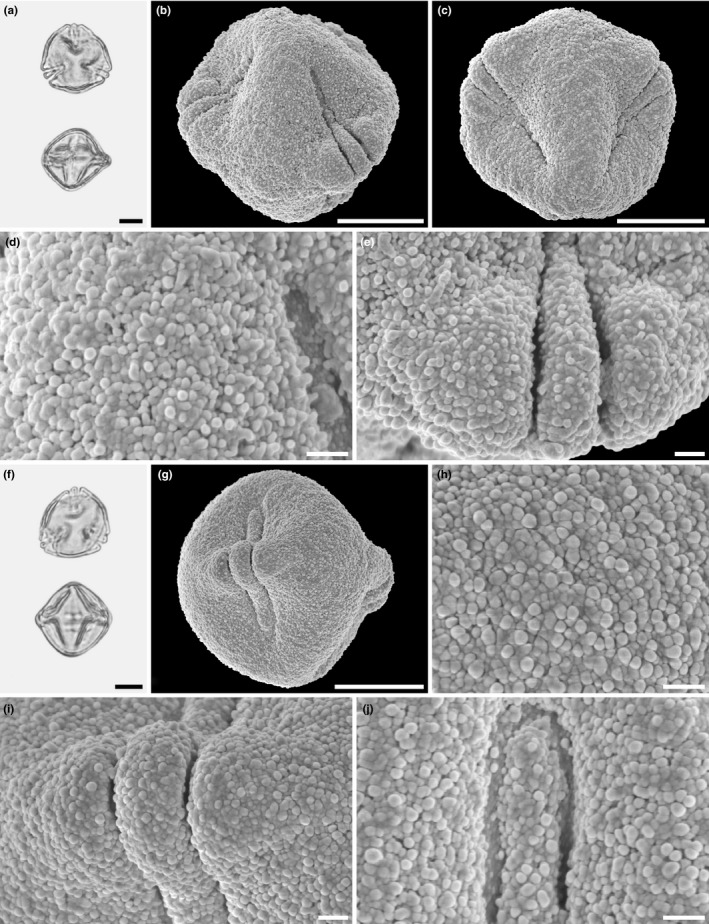
Light microscopy (a, f) and scanning electron microscopy (b–e, g–j) micrographs of fossil *Hagenia* pollen (Mush MT; same grain: a–e; same grain: f–j). (a) Optical cross section in polar (upper) and equatorial (lower) view. (b) Oblique polar view. (c) Oblique polar view. (d) Close‐up of interapertural area. (e) Close‐up of aperture, showing operculum. (f) Optical cross section in polar (upper) and equatorial (lower) view. (g) Equatorial view. (h) Close‐up of interapertural area. (i) Close‐up of aperture, showing operculum. (j) Close‐up of area surrounding operculum in polar region. Scale bars – 10 µm (a–c, f, g), 1 µm (d, e, h–j)

**FIGURE 7:**
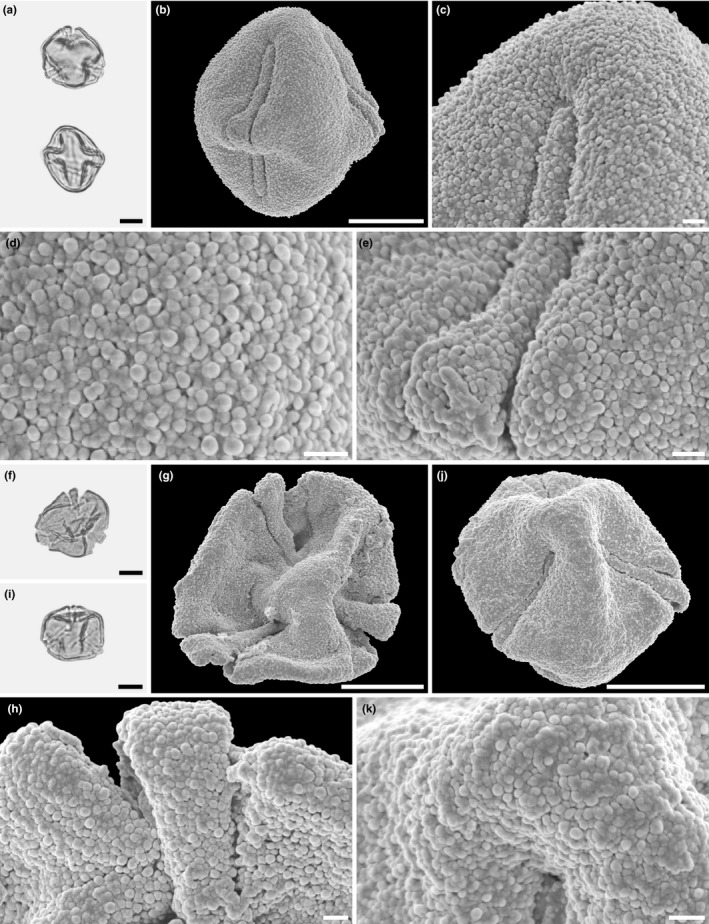
Light microscopy (a, f, i) and scanning electron microscopy (b–e, g, h, j, k) micrographs of fossil *Hagenia* pollen (Mush MT; same grain: a–e; same grain: f–h; same grain: i–k). (a) Optical cross section in polar (upper) and equatorial view (lower). (b) Equatorial view. (c) Close‐up of polar area. (d) Close‐up of interapertural area. (e) Close‐up of aperture, showing operculum. (f) Polar view, pollen flattened. (g) Polar view, pollen flattened. (h) Close‐up of aperture, showing operculum. (i) Polar view, pollen infolded. (j) Oblique polar view. (k) Close‐up of polar area. Scale bars – 10 µm (a, b, f, g, i, j), 1 µm (c–e, h, k)

**FIGURE 8:**
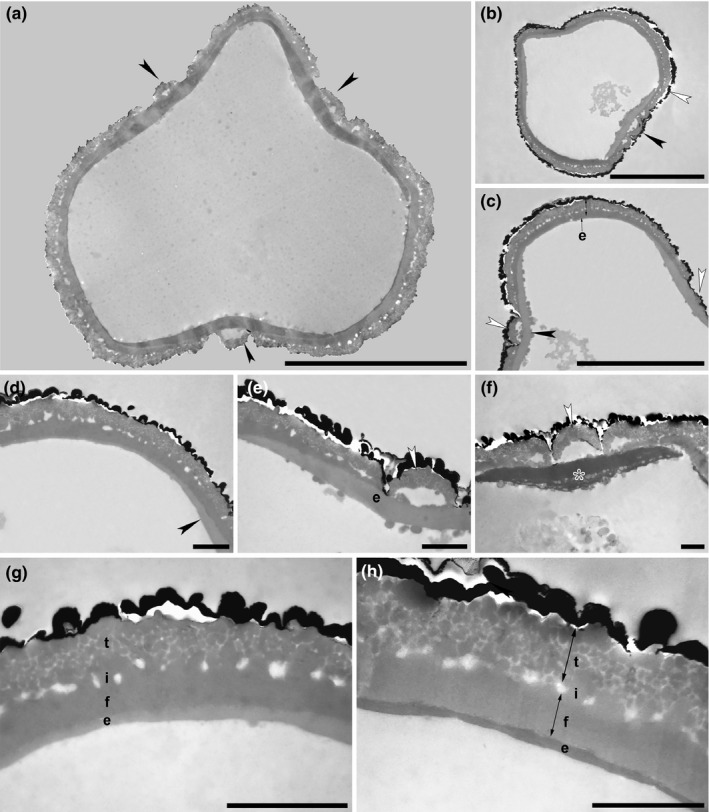
Transmission electron microscopy micrographs of fossil *Hagenia* pollen (Mush MT). (a) Cross section, oblique polar view, showing three operculate apertures (arrowheads), KMnO_4_. (b) Cross section, oblique polar view, showing an operculate aperture (black arrowhead), outermost gold layer (white arrowhead), U + Pb. (c) Detail of pollen wall with aperture (white arrowheads) and interapertural area (arrows), the endexine (e) thickens toward the aperture (black arrow), polar view, U + Pb. (d) Cross section showing interapertural area and thickening of endexine toward aperture (arrowhead), polar view, U + Pb. (e) Transition zone between aperture and interapertural area, aperture (white arrowhead) with thick endexine (e), polar view, U + Pb. (f) Cross section of aperture showing thick endexine (asterisk) and operculum (white arrowhead), polar view, KMnO_4_. (g–h) Cross section of interapertural area showing eutectate tectum (t, arrow), columellate infratectum (i), compact‐continuous foot layer (f, arrow), compact‐continuous endexine (e), and outermost gold cover (electron dense), U + Pb (g) and KMnO_4_ (h). Abbreviations: U + Pb, uranyl acetate/lead citrate; KMnO_4,_ potassium permanganate. Scale bars – 10 µm (a–c), 1µm (d–h)

Pollen, monad, P/E ratio suboblate to prolate, shape convex‐triangular dipyramid, convex triangular in polar view, elliptic to circular or rhombic in equatorial view; equatorial diameter 26.9–32.7 µm in LM, 21.2–30.8 µm in *SEM*, polar axis 25–36.5 µm in LM, 24.8–31.9 µm in *SEM*; tricolporate, aperture protruding, operculate, operculum 17.3–25 µm long in LM, 15.7–22.5 µm long in *SEM*, 2.3–5 µm wide in LM, 2.2–5.1 µm wide in *SEM*; exine 1.7–2.7 µm thick, nexine thinner than sexine (LM); tectate, columellate (*SEM*); sculpture psilate in LM, nanogemmate, nanoclavate, and nanoechinate in *SEM* (Figures 6, 7, 8; Tables 2 and 4).

The ultrastructure (TEM) of the fossil is similar to that of extant *H. abyssinica* pollen. The pollen wall constitutes a sporopollenin, thick structured tectate‐columellate ektexine, and a thin monolayered compact‐continuous endexine. The ektexine is between 1.0 and 1.07 µm thick, with thick continuous tectum (eutectate), columellate infratectum (with short columellae), and thick compact‐continuous foot layer. The internal tectum is granular, composed of small homogeneous elements. The compact endexine forms a thin (about 0.07 µm) continuous layer, increasing in thickness toward the aperture (electron dense). The operculum, formed by the ektexine, covers most of the aperture.

##### Remarks

This is the first fossil *Hagenia* pollen documented using combined LM and *SEM*, and is the first time to be studied with TEM. The pollen morphology (LM and *SEM*) and ultrastructure (TEM) of the fossil grains are similar to those of *H. abyssinica* described above. The fossil and extant pollen grains are compared in Table 4 and below.

### 3.2 Extant versus fossil *Hagenia* pollen

The pollen of *Hagenia abyssinica* and the fossil grains from the Mush Valley site are similar in LM and *SEM* morphology as well as TEM ultrastructure. When comparing the extant and fossil pollen, it is clear they are closely related and of the same genus; however, they exhibit some subtle differences. In equatorial view, pollen of *H. abyssinica* are generally suboblate to subprolate, and most appear to be isodiametric (polar axis ± equal to the equatorial diameter) or slightly oblate when studied with LM. The fossil Mush MT pollen are suboblate to prolate and usually more often distinctly prolate than not. The main difference separating the fossil grains from the extant pollen is in the sculpture as seen with *SEM*. Pollen of *H. abyssinica* are distinctly rugulate (for example see Figures 3e, 4e) at interapertural areas and with nanogemmate/clavate/echinate suprasculpture in *SEM*. These well‐defined rugulae of the extant pollen are not observed in any of the numerous fossil grains studied from the Mush Valley site (for example see Figures 6g, h, 7b, d). Most of the pollen surfaces in the fossil pollen grains are homogenous from the polar regions toward the equator. The nanogemmate/clavate/echinate *SEM* sculpture observed in the fossil grains is the basal sculpture and not identified as suprasculpture in any of the studied grains.

### 3.3 Anthers and dehiscence mode of *Hagenia abyssinica*

**FIGURE 9:**
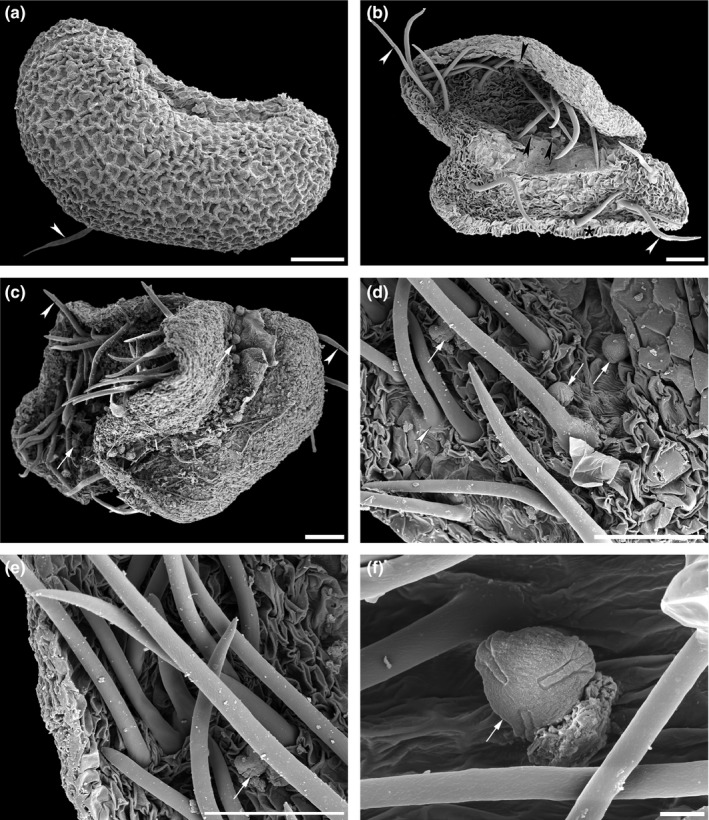
Scanning electron micrographs showing anther dehiscence and occurrence of anther hairs in *Hagenia abyssinica*. (a) Closed anther, ventral side, visible anther hair (arrowhead) indicates beginning of dehiscence (from Burundi, coll. J. Lewalle, 5,739 [MO]). (b) Lateral dehisced anther showing open anther with anther hairs (white arrowhead) and few remaining pollen (black arrowheads); asterisk indicates endothecium (from Tanzania, coll. A.S. MKeya, 922 [MO]). (c–f) Dehisced anthers (oblique views) and close‐ups showing anther hairs (white arrowheads) and remaining pollen (arrows) inside open anthers (from Malawi, coll. I.F. LaCroix, 4,822 [MO]). Scale bars – 100 µm (a–e), 10 µm (f)

Under the dissecting microscope, the male flowers of *H. abyssinica* are eye‐catchingly hairy. Although hairs are abundant on the calyx lobes, the stamens are glabrous (Figure 9a–c; Table 1). The stamens are filamentous and the small anthers (about 0.6 mm) produce numerous pollen. The anthers dehisce along the longitudinal stomial furrow, dividing the thecae into two equal halves thereby releasing the pollen monads (Figure 9b,c). *Hagenia abyssinica* pollen is nearly free of pollen coatings (pollenkitt) and mature pollen grains seem to be only slightly sticky due to remnants from the tapetum and/or locular fluid. In open anthers, thick rounded epidermal unicellular anther hairs are present (Figure 9b–f). Inside dehisced anthers only few pollen grains remain among anther hairs (Figure 9d–f). Ubisch bodies on the inner anther wall are absent (Figure 9f), and starch was not detected (using iodine).

### 3.4 Molecular dating analysis

**FIGURE 10:**
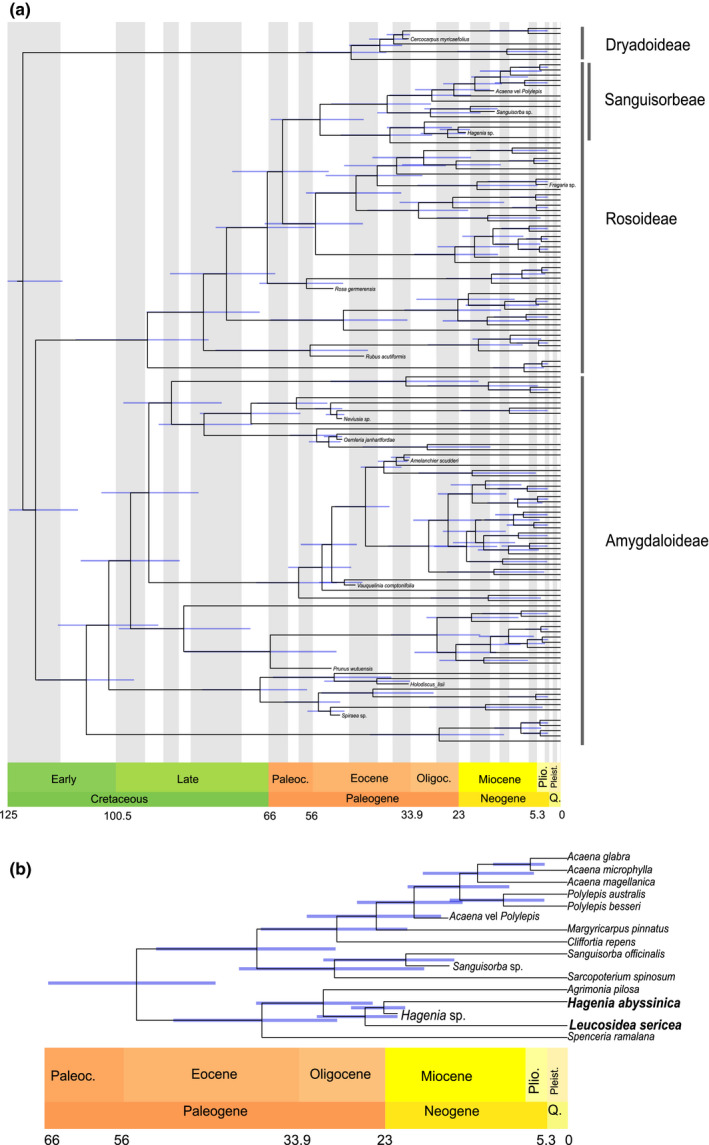
(a) Chronogram obtained from our dating analysis of Rosaceae. (b) Detail showing only the tribe Sanguisorbeae. 95% Higher Posterior Density for ages are shown as blue bars

Our molecular dating analysis retrieves an Early Cretaceous age for the crown group Rosaceae (125–113 Ma 95% Highest Posterior Density, HPD) (Figure 10). The crown group Rosoideae is inferred to have originated not long after, between the Early and Late Cretaceous (110–80 Ma). The tribe Agrimonieae (Sanguisorbeae) is inferred to have originated in the Palaeogene (65–44 Ma), with the Agrimoniinae and Sanguisorbinae originating almost contemporaneously (the former 50–29 Ma and the latter 52–29 Ma). The split between *Hagenia* and *Leucosidea* is inferred to have happened between the early Oligocene and early Miocene (32–21 Ma).

## 4 DISCUSSION

### 4.1 The fossil record of *Hagenia*

The Mush Valley pollen grains presented herein represent the only *Hagenia* fossil pollen documented and illustrated using combined LM and *SEM*. There are currently no macrofossils known that can be attributed to this genus. There is an older pollen occurrence reported from the Hauga section of the late Oligocene Chilga site (27.36 ± 11; Kappelman et al., 2003) in northwestern Ethiopia (Yemane et al. (1985), Yemane et al. (1987); Bonnefille, 2010). Nevertheless, this identification is unaccompanied by a description or micrographs. Also, an examination of pollen samples for this study collected from an adjacent section at Chilga, and certainly of equivalent age, revealed no *Hagenia* pollen. According to Yemane et al. (1985), Yemane et al. (1987), *Hagenia* occurs at less than 5% abundance intermittently among pollen samples from the Hauga section spanning approximately one million years of deposition. The paleoenvironment is reconstructed as dense forest (“swamp forest with temperate trees”; Bonnefille, 2010). Other Afromontane endemics reported include *Hallea rubrostipulata, Macaranga,* and *Afrocrania*, (also not described nor illustrated), but *Podocarpus* and *Juniperus* are absent (Yemane et al., 1985, 1987). *Hagenia* pollen does not occur again in the African fossil record until the Pliocene hominid sites Hadar and Gona (Figure 1) in the Afar depression of Ethiopia (based on LM observation). At Hadar (3.4–2.95 Ma), *Hagenia* pollen co‐occurs in samples with pollen from other high‐elevation, humid forest taxa including *Myrica, Ilex,* and *Olea* (Bonnefille et al., 1987, 2004), all of which are interpreted to have blown in from the neighboring highlands (Bonnefille, 2010). The samples at Gona (~2.6 Ma) are dominated by Afromontane vegetation elements, particularly pollen of *Podocarpus*, and *Hagenia* makes up as much as 6.4% of pollen grains (López‐Sáez & Domínguez‐Rodrigo, 2009). The authors conclude that dry montane forests thrived close to Gona. *Hagenia* pollen has also been reported from the Pleistocene localities Gadeb (2.5–2.3 Ma, Ethiopia; Bonnefille, 1983), Omo Valley (2.5–2 Ma, Ethiopia; Bonnefille, 1976b), Olduvai (~1.8–1.2 Ma, Tanzania; Bonnefille, 1984), Melka Kunture (1.8–0.6 Ma, Ethiopia; Bonnefille et al., 2018), Koobi Fora (~1.6–1.5 Ma, Kenya; Bonnefille, 1976a), and Peninj (1.5–1.35 Ma, Tanzania; Dominguez‐Rodrigo et al., 2001) (all based on LM observations; Figure 1). In all cases, *Hagenia* pollen makes up only a small component of the palynoflora and is thought to be allochthonous and transported in by wind or river. Only Gadeb is above 2000 m in elevation, and this site is reconstructed as dense Ericaceae vegetation, typical of a high‐elevation environment. After Melka Kunture, *Hagenia* pollen is not known from Ethiopia until the late Pleistocene (Figure 1), ~16,700 years ago (Bale Mountains record; Umer et al., 2007), but is found elsewhere in high‐elevation core samples from Kenya, Uganda, Burundi, and Tanzania dating to as early as the last glacial interval, which reaches back beyond the range of radiocarbon dating. Age estimates of 60,000–42,000 years ago for samples from Maundi Crater, Mt. Kilimanjaro (Schüler et al., 2012), from prior to the last glacial maximum (at about 32,000–22,000) for samples from Sacred Lake, Mt. Kenya (Olago et al., 1999), from > 40,000 B.P. in samples from the Uluguru Mtns (where it is absent today; Finch et al., 2009), and from Rusaka, Burundi (Bonnefille et al., 1995), suggest that *Hagenia* was a component of montane forests during appropriate climatic conditions in the Quaternary highlands of East Africa for as far back as records go (Bonnefille & Riollet, 1988; Olago et al., 1999; Schüler et al., 2012; Taylor, 1990).

### 4.2 Phylogenetic relevance

The presence of *Hagenia* pollen at the early Miocene Mush Valley site is relevant to the evolutionary history of the genus, which has been assessed in recent phylogenetic studies of the Rosaceae by Xiang et al. (2017) and Zhang et al. (2017). Xiang et al. (2017) provide dates for the origin of the subfamily Rosoideae at about 99 Ma, and the age of the split between tribes Agrimonieae and Potentilleae by the middle Paleocene at about 63 Ma. *Hagenia* itself is reported to have split from its sister taxon *Leucosidea* at an estimated date of 18 Ma (Xiang et al., 2017). The age of the Mush Valley samples is 22.63–21.73 Ma, which makes the occurrence of its pollen there inconsistent with the age provided by Xiang et al. (2017) for the origin of the genus. The time difference between the hypothesized origin of the tribe Agrimonieae at 63 Ma and the occurrence of *Hagenia* fossils at 22.63–21.73 Ma, though leaves more than 40 million years for the evolution of genera and species currently recognized within the tribe. Zhang et al. (2017) provide a much younger date for the origin of the Agrimonieae at 36.13 (35.07–39.58 highest posterior density), which provides approximately 15 million years for the evolution of the tribe. Our analysis (Figure 10), though retrieving ages for the Agrimonieae consistent with Xiang et al. (2017), suggests an older age for the *Hagenia*‐*Leucosidea* split thanks to the integration of the new *Hagenia* fossil pollen. However, the split is still relatively young, with the youngest 95% HPD boundary in the Aquitanian.

### 4.3 Ecological preferences of fossil and extant *Hagenia*

The discovery of *Hagenia* pollen at Mush tempts one to use its current restricted distribution to infer the ecological setting at Mush, but begs the question, is it appropriate to use the ecological envelope of extant *Hagenia* as a key to the past. Today, *Hagenia* occurs predominantly at elevations from 2,000–3,000 m, within the “Afromontane region” or “Afromontane archipelago‐like regional center of endemism” (White, 1983) of Central and East Africa. As an upland species *H. abyssinica* could potentially provide information about paleoaltitude that is otherwise lacking. However, paleoaltitude must be estimated from a proxy independent of plant remains because (1) fossil plants may have had different ecological tolerances from their living counterparts, and (2) even if ancient and modern tolerances are equal, warmer or cooler past climates will influence the elevation at which plants grew when they were fossilized. Thus, we use other proxies here to estimate roughly the paleoaltitude at the Mush site, and review prior conclusions about the local plant community and climate around the paleolake. Those data inform, as much as possible, our understanding of *Hagenia* ecology at Mush, rather than taking the reverse approach of using living *Hagenia* to understand Mush paleoecology.

Today, the Mush Valley locality is at an elevation of 2,680 m, on the northwestern Plateau near the main Ethiopian Rift escarpment. The greatest volume of the Ethiopian Plateau consists of late Oligocene flood basalts, which top out at about 2000 m. Higher elevations on the Plateau occur in association with Miocene volcanism; the Mush Valley paleolake itself formed as a consequence of an active shield volcano, Guguftu, nearly 22 Ma (Kieffer et al., 2004). There are approximately 700 m thick layers of volcanic and interspersed clastic deposits related to the Guguftu volcano, directly overlying basalt traps of an imprecisely known paleoelevation. The consensus opinion from a variety of studies indicates deposition into the Nile Delta of sediments originating on the Ethiopian Plateau beginning in the late Oligocene nearly contemporaneously with massive flood basalt eruptions, indicating a topographic high relative to the delta region (Faccenna et al., 2019; Fielding et al., 2018; Ismail & Abdelsalam, 2012; Macgregor, 2012; Pik et al., 2003; Sembroni et al., 2016). Most studies, whether using remote sensing coupled with nickpoint geomorphology or thermochronometry (e.g., U‐Th/He), conclude that the Blue Nile has been the main, if not only, source of sedimentation to the Nile Basin since the late Oligocene, and additional episodes of elevation took place between about 22 and 10 Ma (Pik et al., 2003; Sembroni et al., 2016; Fielding et al., 2018; Faccenna et al., 2019). Ismail and Abdelsalam (2012) specify uplift at 22 Ma associated with development of shield volcanoes such as that at Mush (Guguftu). Faccenna et al. (2019) are unique in providing numerical paleoaltitudinal estimates: the Ethiopian Plateau region was 1,800 to 2,200 m at about 40 Ma (prior to flood basalt eruptions), and by 20 Ma the region to southeast of Lake Tana (including the Mush Valley area) was ~ 2,200–2,400 m. If these estimates are accurate, and other variables are held constant (equal to modern), this elevation would be appropriate for the growth of *Hagenia*. Paleoclimate at the Mush locality has so far been documented by calculation of mean annual precipitation from leaf morphology at six stratigraphic horizons, including the specific location of deposits also preserving *Hagenia* pollen (Bush et al., 2017). Precipitation estimates have overlapping margins of error and range from about 1,500–1,600 mm/yr. This is wetter than mean annual precipitation at the site today (~1,100 mm/yr). A mean annual temperature has not yet been reported for the fossil site, but global paleotemperature curves based upon the marine oxygen isotopic record indicate the global mean was higher than modern (Zachos et al., 2008).

Taxonomic and morphologic assessments of leaves, phytoliths, and pollen are consistent with a closed‐canopy, mixed‐moist semi‐evergreen forest with limited palm diversity, and showing botanical affinities with species found today in West, Central, and East Africa (e.g., Bush et al., 2017; Currano et al., 2020; Danehy, 2010; Grímsson et al., 2019; Pan et al., 2012, 2014). Both *Newtonia* and *Tacca* (Pan et al., 2012), found today in moist tropical or secondary forests, and *Sclerosperma* (Currano et al., 2020; Ulrich & Grímsson, 2020), that today grows in tropical lowland rainforests (swamps), were identified at Mush. Moreover, fossil leaf stable carbon isotope (δ^13^C) values match the average for modern African tropical rain forests (Bush et al., 2017), and the 1523–1647 mm/yr reconstructed mean annual precipitation at early Miocene Mush (Bush et al., 2017) is comparable to that of modern mixed‐moist semi‐evergreen rainforests in the Guineo‐Congolian regional center of endemism, one of Africa's eighteen major phytochoria (White, 1979, 1983, 1993).

While the Mush paleoflora is consistent with White’s (1983) closed‐canopy, mixed‐moist semievergreen forest described from the modern Guineo‐Congolian lowlands, geological studies consistently indicate the presence of a mantle plume causing doming (up to 1,200 m [Faccenna et al., 2019]) and flood basalt eruptions since the late Oligocene, followed by additional height in the form of shield volcanoes including Guguftu at Mush, and further uplift sometime between about 20 and 10 million years ago. According to Faccenna et al. (2019), the Mush region would have been as high as 2,400 m. Assuming global temperatures warmer than today, and higher rainfall, the early Miocene Ethiopian Highlands could have accommodated moist forests structurally similar to those found today in the lowland to transitional areas of Central Africa (e.g., the margins of the Albertine Rift). If *Hagenia* was an upland, cool climate, species in the early Miocene as it is today, then perhaps its pollen was transported to the lake basin by long distance downward dispersal from higher elevations. *Hagenia* has not been found among more than 2,400 fossil leaves examined from Mush. None resembles modern *H. abyssinca's* pinnately compound leaves and sessile, oblong leaflets with acuminate apices, rounded to subcordate bases, dentate margins, and regularly, closely spaced and semicraspedodromous secondary veins. Only six leaf types at Mush have toothed margins, and none of these match even two of the additional characters described above.

The Mush palynological assemblage has not currently revealed any other pollen type typical of long‐distance wind dispersal that could have originated from high‐elevation montane forests. Danehy (2010) reported *Olea*, a high‐elevation taxon, from the Mush Valley, but Oleaceae pollen grains are not easily assigned to particular genera without *SEM* studies (e.g., Punt et al., 1991) and can also be confused with pollen from, for example, Euphorbiaceae. Also, Podocarpaceae (*Podocarpus*) and Cupressaceae (*Juniperus*) pollen that are certainly long‐distance wind‐dispersed taxa and typical of modern Afromontane forests have not been observed in any sample from the Mush Valley site (see also Danehy, 2010). It seems to be the general consensus that *Hagenia abyssinica* is a strictly wind‐pollinated species, with the possibility of long‐distance pollen dispersal (e.g., Ayele et al., 2009, 2011; Gichira et al., 2017; Negash, 2010). Interestingly, a recent study by Schüler et al. (2014), on the relation between Afromontane vegetation and pollen rain, shows that *Hagenia* pollen only occurs in pollen traps located within the close vicinity of the parent plant. There is no documentation of *Hagenia* pollen being transported long‐distances downhill into communities where the plant is not growing. Pollen of other wind‐dispersed woody taxa, such as *Podocarpus*, are prominent in pollen traps outside their vegetation belts. In this regard, Schüler et al. (2014) concluded that the presence of dispersed *Hagenia* pollen is likely to be a local signal. This scenario questions if *Hagenia* is really wind‐pollinated and can travel long distances.

Literature reports about the pollination mode of *H. abyssinica* are inconsistent and vary from wind pollination, with the larger female inflorescence supposedly trapping pollen dispersed by wind (Asmare, 2005; Ayele et al., 2009, 2011, 2017; Gichira et al., 2017; Negash, 2010) to pollination by bees, with nectar producing female and male flowers (Negash, 2010, p. 181). Bees have been observed collecting pollen from male flowers and nectar from female flowers (Fichtl & Admasu, 1994; Asmare, 2005; Ayele et al., 2017), and that bees collect pollen and nectar from *H. abyssinica* is also evident from African honey (Legesse, 1995). In this relation, hairs observed on floral leaves of male flowers and dehisced anthers in *H. abyssinica* become relevant (see Results). Floral hairs related to pollination in the androecium is a rare feature in angiosperms, but found in, for example, Commelinaceae (Faden, 1992), *Echium*, *Esterhazya* (Boraginaceae; Hesse et al., 2000), some Fabaceae (Hughes, 1997), and *Salvia verticillata* (Lamiaceae; Ulrich & Tweraser, 2016). Their function is always correlated with pollination by insects and manipulates how they behave on the flower (includes attracting insects, climbing structures, collecting pollen), but also affects pollen release (retaining pollen inside and/or preventing premature pollen release and/or immediate loss) (Castellanos et al., 2005; Faden, 1992; Hesse et al., 2000). Hairy anthers are also supposed to restrict pollen removal by buzz pollination from bee vibration (Castellanos et al., 2005; Vallejo‐Marín, 2019). Staminal hairs can also attract pollinators, for example, with long colored hairs on filaments (e.g., *Tradescantia)* or with staminal hairs contrasting in color with petals (e.g., *Cyanotis*) (Faden, 1992, p. 50). In case of *H. abyssinica,* the stamens are glabrous but according to literature they are colored orange to white (POWO, 2019; Simion, 2018) and might also attract pollinators.

The mode of anther dehiscence also affects the amount of pollen released. Pollen of zoophilous plants usually remains in the open anther after dehiscence and is gradually exposed over time, but, remaining pollen of mainly insect pollinated flowers may also be transported passively by wind when flowers wither and pollen dries out (Willmer, 2011). Usually, anthers open synchronously, releasing all pollen at once (Willmer, 2011). In some of the *SEM*‐investigated anthers (see Results), the slit seemed to split progressively, leading to gradual release of pollen, but without proper field observation, it remains unclear how the anther splits (synchronously or progressively) and also if pollen is deposited onto the hairy calyx lobes (accidental dislocation or secondary presentation). In case of secondary pollen presentation, pollen must be sticky to adhere to other floral organs and subsequent pollinators, but the *Hagenia* pollen is without pollenkitt. Moreover, secondary pollen presentation is rare in angiosperms, confined to 25 families (Howell et al., 1993; Yeo, 1993), and not reported for Rosaceae. It is more likely that *H. abyssinica* pollen is dislocated by wind, visitors, or at full anther dehiscence and is retained among the hairs of the calyx lobes where it is accessible to bees. A changing environment could cause transition from entomophily to anemophily, or even ambophily for reproductive assurance. The colored male and female *H. abyssinica* flowers, the occurrence of anther hairs, the restricted distribution of dispersed pollen, the occurrence of pollen in honey, and the documented visits of bees to both male and female flowers indicate that *H. abyssinica* is not strictly wind‐pollinated. We suggest that *Hagenia* was primarily insect (?bee) pollinated, as indicated by the anther hairs, and over time, in response to environmental pressure gradually changed into ambophily.

The data produced by our study of early Miocene *Hagenia* pollen from the Mush Valley informs our understanding of its evolutionary history and offers possible explanations for its current limited distribution as an East African Highlands endemic. The new phylogenetic analysis indicates the split between *Hagenia* and its sister taxon *Leucosidea* took place around the Oligocene‐Miocene boundary. Thus, the *Hagenia* pollen at Mush derives from an early relative of the living species if not the living species itself. The abundance of *Hagenia* pollen in the paleolake sediments, its likely entomophilous pollination mode, and the rarity of long‐distance transport of *Hagenia* pollen today most likely indicate it was present locally at Mush, although it has not been recognized among the (large) fossil leaf collection. The occurrence of *Hagenia* at Mush does not itself indicate that the site was at a high paleoelevation, although nickpoint and thermochronometric studies indicate drainage to the Nile basin by the late Oligocene and further uplift and volcanism at 22 Ma; Faccenna et al. (2019) suggest the elevation at Mush would have been as high as 2,500. Nevertheless, the paleoflora is interpreted as a closed‐canopy, mixed‐moist semi‐evergreen forest described from the modern Guineo‐Congolian lowlands with average yearly precipitation of about 1500–1600 mm (Bush et al., 2017). These modern forests are similar to (elevationally) transitional forests present today near the flanks of the Albertine Rift, although *Hagenia* does not occur there. Thus, living *Hagenia* are the descendants (or remains) of an ancestral stock that lived in warmer and wetter conditions than its relatives tolerate today. Descendants evolved in post‐mid‐Miocene times, likely in response to environmental pressures related to changes in topography, such as development of the Rift Valley, and climate. It seems that when rift‐related mountain chains in East Africa formed, and the climate in lowland East Africa became drier, *Hagenia* was pushed, giving way for competition, to higher elevations. There, on the island mountains, it survives in comparatively “cold” but mostly humid environments and is an example of niche evolution in response to a complex history of changes in topography related to regional tectonics, and climate.

## CONFLICT OF INTEREST

All authors declare no competing interests.

## AUTHOR CONTRIBUTIONS

**Friðgeir Grímsson:** Conceptualization (lead); Investigation (lead); Methodology (lead); Project administration (lead); Writing‐original draft (lead). **Silvia Ulrich:** Investigation (supporting); Methodology (supporting); Writing‐original draft (supporting). **Mario Coiro:** Investigation (supporting); Methodology (supporting); Writing‐original draft (supporting). **Shirley A Graham:** Investigation (supporting); Writing‐original draft (supporting). **Bonnie F Jacobs:** Conceptualization (supporting); Investigation (supporting); Methodology (supporting); Writing‐original draft (supporting). **Ellen Diane Currano:** Investigation (supporting); Writing‐original draft (supporting). **Alexandros Xafis:** Investigation (supporting); Writing‐original draft (supporting). **Reinhard Zetter:** Conceptualization (supporting); Investigation (supporting); Methodology (supporting); Writing‐original draft (supporting).

## Supporting information

Figure S1

Supplementary Material

## Data Availability

Data (consensus tree and nexus alignment) are available through Figshare (https://doi.org/10.6084/m9.figshare.13728655).

## References

[ece37408-bib-0001] AFT (2009). The Agroforestree Database. http://worldagroforestry.org/output/agroforestree‐database. Accessed July 2019.

[ece37408-bib-0002] Asmare, K. (2005). Estimation of sex‐related genetic diversity of Hagenia abyssinica (Bruce) J.F.Gmel using random amplified polymorphic DNA (RAPD) markers. MS. M.Sc. thesis, Addis Ababa University, Ethiopia. http://localhost:80/xmlui/handle/123456789/5870

[ece37408-bib-0003] Assefa, B. , Glatzel, G. , & Buchmann, C. (2010). Ethnomedicinal uses of *Hagenia abyssinica* (Bruce) J.F. Gmel. Among rural communities of Ethiopia. Journal of Ethnobiology and Ethnomedicine, 6, 20. 10.1186/1746-4269-6-20 20701760 PMC2928183

[ece37408-bib-0004] Ayele, T. B. , Gailing, O. , & Finkeldey, R. (2011). Assessment and integration of genetic, morphological and demographic variation in *Hagenia abyssinica* (Bruce) JF Gmel to guide its conservation. Journal for Nature Conservation, 19(1), 8–17. 10.1016/j.jnc.2010.03.001

[ece37408-bib-0005] Ayele, T. B. , Gailing, O. , & Finkeldey, R. (2017). Spatial distribution of genetic diversity in populations of *Hagenia abyssinica* (Bruce) J.F Gmel from Ethiopia. *Annals of* . Forest Research, 60(1), 47–62. 10.15287/afr.2016.740

[ece37408-bib-0006] Ayele, T. B. , Gailing, O. , Umer, M. , & Finkeldey, R. (2009). Chloroplast DNA haplotype diversity and postglacial recolonization of *Hagenia abyssinica* (Bruce) J.F. Gmel. Ethiopia. Plant Systematics and Evolution, 280, 175–185. 10.1007/s00606-009-0177-5

[ece37408-bib-0008] Bekele, G. , & Reddy, R. (2014). Folklore medicinal uses of *Hagenia abyssinica* (Bruce) J.F. Gmel to treat human ailments among the communities of Abaya district, Borana zone, Oromia regional state Ethiopia. International Journal of Physical and Social Sciences, 4(12), 394–408.

[ece37408-bib-0009] Bombosi, P. (2016). Agrimonia eupatoria . In: PalDat – A palynological database. https://www.paldat.org/pub/Agrimonia_eupatoria/301307. Accessed December 13, 2019.

[ece37408-bib-0010] Bonnefille, R. (1976a). Implications of pollen assemblage from Koobi Fora Formation, East Rudolf, Kenya. Nature, 264, 403–407.

[ece37408-bib-0011] Bonnefille, R. (1976b). Palynological evidence for an important change in the vegetation of the Omo basin between 2.5 and 2 million years ago. In Y. Coppens , F. C. Howell , G. L. Isaac , & R. E. F. Leakey (Eds.), Earliest man and environment in the Lake Rudolf Basin (pp. 421–431). University of Chicago Press.

[ece37408-bib-0012] Bonnefille, R. (1983). Evidence for a cooler and drier climate in the Ethiopian uplands towards 2.5 myr ago. Nature, 303, 487–491. 10.1038/303487a0

[ece37408-bib-0013] Bonnefille, R. (1984). Palynological research at Olduvai Gorge. National Geographic Society Research Reports, 17, 227–243.

[ece37408-bib-0014] Bonnefille, R. (2010). Cenozoic vegetation, climate changes and hominid evolution in tropical Africa. Global and Planetary Change, 72(4), 390–411. 10.1016/j.gloplacha.2010.01.015

[ece37408-bib-0015] Bonnefille, R. , Melis, R. T. , & Mussi, M. (2018). Variability in the mountain environment at Melka Kunture archaeological site, Ethiopia, during the early Pleistocene (similar to 1.7 Ma) and the mid‐Pleistocene transition (0.9‐0.6 Ma). In: R. Gallotti , & M. Mussi (Eds). The Emergence of the Acheulean in East Africa and Beyond. Vertebrate Paleobiology and Paleoanthropology. : Springer, 93–114. 10.1007/978-3-319-75985-2_5

[ece37408-bib-0016] Bonnefille, R. , Potts, R. , Chalie, F. , Jolly, D. , & Peyron, O. (2004). High‐resolution vegetation and climate change associated with Pliocene *Australopithecus afarensis* . Proceedings of the National Academy of Sciences of the United States of America, 101(33), 12125–12129. 10.1073/pnas.0401709101 15304655 PMC514445

[ece37408-bib-0017] Bonnefille, R. , & Riollet, G. (1988). The Kashiru pollen sequence (Burundi) palaeoclimatic implications for the last 40,000 yr BP in tropical Africa. Quaternary Research, 30(1), 19–35. 10.1016/0033-5894(88)90085-3

[ece37408-bib-0018] Bonnefille, R. , Riollet, G. , Buchet, G. , Icole, M. , Lafont, R. , & Arnold, M. (1995). Glacial/interglacial record from intertropical Africa, high resolution pollen and carbon data at Rusaka, Burundi. Quaternary Science Reviews, 14, 917–936. 10.1016/0277-3791(95)00071-2

[ece37408-bib-0019] Bonnefille, R. , Vincens, A. , & Buchet, G. (1987). Palynology, stratigraphy and paleoenvironment of a Pliocene hominid site (2.9‐3.3 my) at Hadar, Ethiopia. Palaeogeography, Palaeoclimatology, Palaeoecology, 60, 249–281. 10.1016/0031-0182(87)90035-6

[ece37408-bib-0020] Brown, J. W. , Walker, J. F. , & Smith, S. A. (2017). Phyx: Phylogenetic tools for unix. Bioinformatics, 33(12), 1886–1888. 10.1093/bioinformatics/btx063 28174903 PMC5870855

[ece37408-bib-0021] Bush, R. T. , Wallace, J. , Currano, E. D. , Jacobs, B. F. , McInerney, F. A. , Dunn, R. E. , & Tabor, N. J. (2017). Cell anatomy and leaf δ^13^ C as proxies for shading and canopy structure in a Miocene forest from Ethiopia. Palaeogeography, Palaeoclimatology, Palaeoecology, 485, 593–604. 10.1016/j.palaeo.2017.07.015

[ece37408-bib-0022] Bussmann, R. W. (2006). Vegetation zonation and nomenclature of African mountains‐an overview. Lyonia, 11(1), 41–66.

[ece37408-bib-0023] Castellanos, M. C. , Wilson, P. , Keller, S. J. , Wolfe, A. D. , & Thomson, J. D. (2005). Anther evolution: Pollen presentation strategies when pollinators differ. The American Naturalist, 167(2), 288–296. 10.1086/498854 16670987

[ece37408-bib-0024] Chung, K.‐S. , Elisens, W. J. , & Skvarla, J. J. (2010). Pollen morphology and its phylogenetic significance in tribe Sanguisorbeae (Rosaceae). Plant Systematics and Evolution, 285, 139–148. 10.1007/s00606-009-0262-9

[ece37408-bib-0025] Coiro, M. , Doyle, J. A. , & Hilton, J. (2019). How deep is the conflict between molecular and fossil evidence on the age of angiosperms? New Phytologist, 223(1), 83–99. 10.1111/nph.15708 30681148

[ece37408-bib-0026] Couvreur, T. L. P. , Chatrou, L. W. , Sosef, M. S. M. , & Richardson, J. E. (2008). Molecular phylogenetics reveal multiple tertiary vicariance origins of the African rain forest trees. BMC Biology, 6, 54. 10.1186/1741-7007-6-54 19087283 PMC2628871

[ece37408-bib-0027] Currano, E. D. , Jacobs, B. F. , Bush, R. T. , Novello, A. , Feseha, M. , Grímsson, F. , McInerney, F. A. , Michel, L. , Pan, A. D. , Phelps, S. , Polissar, P. , Strömberg, C. A. E. , & Tabor, N. J. (2020). Ecological dynamic equilibrium in an early Miocene (21.7 Ma) forest. Ethiopia. Palaeogeography, Palaeoclimatology, Palaeoecology, 539, 109425. 10.1016/j.palaeo.2019.109425

[ece37408-bib-0028] Danehy, D. R. (2010). Terrestrial vegetation reconstructions spanning the Paleogene – Neogene boundary in the Ethiopian highlands. MS. Dissertation. : Southern Methodist University, pp. 174

[ece37408-bib-0029] Denk, T. , Grímsson, F. , Zetter, R. , & Símonarson, L. A. (2011). Late Cainozoic floras of Iceland – 15 million years of vegetation and climate history in the northern North Atlantic (p. 854): Springer, Netherlands. 10.1007/978-94-007-0372-8

[ece37408-bib-0030] Dominguez‐Rodrigo, M. , Lopez‐Saez, J. A. , Vincens, A. , Alcala, L. , Luque, L. , & Serrallonga, J. (2001). Fossil pollen from the Upper Humbu Formation of Peninj (Tanzania): Hominid adaptation to a dry open Plio‐Pleistocene savanna environment. Journal of Human Evolution, 40(2), 151–157. 10.1006/jhev.2000.0440 11161958

[ece37408-bib-0031] Durango de Cabrera, J. , & Vergel, M. M. (1989). Contribución al conocimiento de las hojas de Fagaceae de la Formación Cullen, Terciario del Territorio Nacional de Tierra del Fuego, República Argentina. Acta Geológica Lilloana, 17(1), 67–73.

[ece37408-bib-0032] Edelman, D. W. (1975). The Eocene Germer Basin Flora of South‐Central Idaho [MS Thesis] (p. 142). University of Idaho.

[ece37408-bib-0033] Eriksson, T. , Hibbs, M. S. , Yoder, A. D. , Delwiche, C. F. , & Donoghue, M. J. (2003). The phylogeny of Rosoideae (Rosaceae) based on sequences of the internal transcribed spacers (ITS) of nuclear ribosomal DNA and the *trn*L/F region of chloroplast DNA. International Journal of Plant Sciences, 164(2), 197–211. 10.1086/346163

[ece37408-bib-0034] Faccenna, C. , Glišović, P. , Forte, A. , Becker, T. W. , Garzanti, E. , Sembroni, A. , & Gvirtzman, Z. (2019). Role of dynamic topography in sustaining the Nile River over 30 million years. Nature Geoscience, 12, 1012–1017. 10.1038/s41561-019-0472-x

[ece37408-bib-0035] Faden, R. B. (1983). Phytogeography of African Commelinaceae. Bothalia, 14(3–4), 553–557. 10.4102/abc.v14i3/4.1207

[ece37408-bib-0036] Faden, R. B. (1992). Floral attraction and floral hairs in the Commelinaceae. Annals of the Missouri Botanical Garden, 79(1), 46–52. 10.2307/2399808

[ece37408-bib-0037] Fetene, M. , & Feleke, Y. (2001). Growth and photosynthesis of seedlings of four tree species from a dry tropical afromontane forest. Journal of Tropical Ecology, 17(2), 269–283.

[ece37408-bib-0038] Fichtl, R. , & Admassu, A. (1994). Honeybee flora of Ethiopia (p. 510). Margraf Verlag.

[ece37408-bib-0039] Fielding, L. , Najman, R. , Millar, I. , Butterworth, P. , Garzanti, E. , Vezzoli, G. , Barfod, D. , & Kneller, B. (2018). The initiation and evolution of the River Nile. Earth and Planetary Science Letters, 489, 166–178. 10.1016/j.epsl.2018.02.031

[ece37408-bib-0040] Finch, J. , Leng, M. J. , & Marchant, R. (2009). Late Quaternary vegetation dynamics in a biodiversity hotspot, the Uluguru Mountains of Tanzania. Quaternary Research, 72(1), 111–122. 10.1016/j.yqres.2009.02.005

[ece37408-bib-0041] Friis, I. (1992). Forests and forest trees of northeast tropical Africa: their natural habitats and distribution patterns in Ethiopia, Djibouti and Somalia. Kew Bulletin Additional Series XV: 1‐396, London, UK: HMSO.

[ece37408-bib-0042] Garcia‐Massini, J. L. , Zamaloa, M. C. , & Romero, E. J. (2004). Fungal fruiting bodies in the Cullen Formation (Miocene) in Tierra del Fuego, Argentina. Ameghiniana, 41(1), 83–90.

[ece37408-bib-0043] Gichira, A. W. , Li, Z. , Saina, J. K. , Long, Z. , Hu, G. , Gituru, R. W. , Wang, Q. , & Chen, J. (2017). The complete chloroplast genome sequence of an endemic monotypic genus *Hagenia* (Rosaceae): Structural comparative analysis, gene content and microsatellite detection. PeerJ, 5, e2846. 10.7717/peerj.2846 28097059 PMC5228516

[ece37408-bib-0044] Grímsson, F. , & Denk, T. (2007). Floristic turnover in Iceland from 15 to 6 Ma – extracting biogeographical signals from fossil floral assemblages. Journal of Biogeography, 34(9), 1490–1504. 10.1111/j.1365-2699.2007.01712.x

[ece37408-bib-0045] Grímsson, F. , Denk, T. , & Símonarson, L. A. (2007a). Middle Miocene floras of Iceland – the early colonization of an island? Review of Palaeobotany and Palynology, 144(3–4), 181–219. 10.1016/j.revpalbo.2006.07.003

[ece37408-bib-0046] Grímsson, F. , Denk, T. , & Zetter, R. (2008). Pollen, fruits, and leaves of *Tetracentron* (Trochodendraceae) from the Cainozoic of Iceland and western North America and their palaeobiogeographic implications. Grana, 47(1), 1–14. 10.1080/00173130701873081

[ece37408-bib-0047] Grímsson, F. , Graham, S. A. , Coiro, M. , Jacobs, B. F. , Xafis, A. , Neumann, F. , Scott, L. , Sakala, J. , Currano, E. D. , & Zetter, R. (2019). Origin and divergence of Afro‐Indian Picrodendraceae: Linking pollen morphology, dispersal modes, fossil records, molecular dating and paleogeography. Grana, 58(4), 227–275. 10.1080/00173134.2019.1594357 31275086 PMC6582451

[ece37408-bib-0048] Grímsson, F. , Grimm, G. W. , & Zetter, R. (2017). Tiny pollen grains: First evidence of Saururaceae from the Late Cretaceous of western North America. PeerJ, 5, e3434. 10.7717/peerj.3434 28626610 PMC5472062

[ece37408-bib-0049] Grímsson, F. , Grimm, G. W. , & Zetter, R. (2018). Evolution of pollen morphology in Loranthaceae. Grana, 57, 16–116. 10.1080/00173134.2016.1261939 29386990 PMC5771552

[ece37408-bib-0050] Grímsson, F. , & Símonarson, L. A. (2008a). Iceland’s ancient forests. Skógræktarritið 2008/2: 14–30. (in Icelandic)

[ece37408-bib-0051] Grímsson, F. , & Símonarson, L. A. (2008b). Upper Tertiary non‐marine environments and climatic changes in Iceland. Jökull, 58, 303–314.

[ece37408-bib-0052] Grímsson, F. , Símonarson, L. A. , & Denk, T. (2007b). Late Langhian to early Serravallian floras of Iceland. Náttúrufræðingurinn, 75(2–4), 85–106. (in Icelandic).

[ece37408-bib-0053] Habtemariam, A. A. , & Woldetsadik, A. M. (2019). Current distribution, regeneration and management practice of *Hagenia abyssinica* in different agroforestry systems of Ethiopia: A review. International Journal of Biodiversity and Conservation, 11(9), 266–271. 10.5897/IJBC2019.1294

[ece37408-bib-0054] Halbritter, H. , Ulrich, S. , Grímsson, F. , Weber, M. , Zetter, R. , Hesse, M. , Buchner, R. , Svojtka, M. , & Frosch‐Radivo, A. (2018). Illustrated pollen terminology, 2nd ed. (p. 483). Springer. 10.1007/978-3-319-71365-6

[ece37408-bib-0055] Hardarson, B. S. , Fitton, J. G. , Ellam, R. M. , & Pringle, M. S. (1997). Rift relocation – a geochemical and geochronological investigation of a palaeo‐rift in northwest Iceland. Earth and Planetary Science Letters, 153(3–4), 181–196. 10.1016/S0012-821X(97)00145-3

[ece37408-bib-0056] Hayat, M. A. (2000). Principles and techniques of electron microscopy, 4th ed. (p. 564). Cambridge University Press.

[ece37408-bib-0057] Hedberg, O. (1989). Rosaceae. In: I. Hedberg , & S. Edwards (Eds.), Flora of Ethiopia, Pittosporaceae to Araliaceae, Vol. 3. : National Herbarium, Addis Ababa. 10.1111/j.1756-1051.1992.tb01322.x

[ece37408-bib-0058] Hesse, M. , Vogel, S. , & Halbritter, H. (2000). In A. Dafni , M. Hesse , & E. Pacini (Eds.), Thread‐forming structures in angiosperm anthers: Their diverse role in pollination ecology (p. 341). Pollen and Pollination. Springer. 10.1007/978-3-7091-6306-1_15

[ece37408-bib-0059] Howell, G. J. , Slater, A. T. , & Knox, R. B. (1993). Secondary pollen presentation in angiosperms and its biological significance. Australian Journal of Botany, 41(5), 417–438. 10.1071/BT9930417

[ece37408-bib-0060] Hughes, C. E. (1997). Variation in anther and pollen morphology in *Leucena* Benth. (Leguminosae‐Mimosoideae). Botanical Journal of the Linnean Society, 123(3), 177–196.

[ece37408-bib-0061] Ismail, E. H. , & Abdelsalam, M. (2012). Morpho‐tectonic analysis of the Tekeze River and the Blue Nile drainage systems on the Northwestern Plateau, Ethiopia. Journal of African Earth Sciences, 69, 34–47. 10.1016/j.jafrearsci.2012.04.005

[ece37408-bib-0062] Jiang, W. , He, H.‐J. , Lu, L. , Burgess, K. S. , Wang, H. , & Li, D.‐Z. (2019). Evolution of angiosperm Pollen. 7. Nitrogen‐Fixing Clade. Annals of the Missouri Botanical Garden, 104(2), 171–229. 10.3417/2019337

[ece37408-bib-0063] Jima, T. T. (2018). Medicinal plants used in the treatment of livestock diseases in Berbere district of Bale zone, Oromia region, Ethiopia. Journal of Medicinal Plants Research, 12(20), 270–277. 10.5897/JMPR2018.6598

[ece37408-bib-0064] Kappelman, J. , Rasmussen, D. T. , Sanders, W. J. , Feseha, M. , Bown, T. , Copeland, P. , Crabaugh, J. , Fleagle, J. , Glantz, M. , Gordon, A. , Jacobs, B. , Maga, M. , Muldoon, K. , Pan, A. , Pyne, L. , Richmond, B. , Ryan, T. , Seiffert, E. R. , Sen, S. , … Winkler, A. (2003). Oligocene mammals from Ethiopia and faunal exchange between Afro‐Arabia and Eurasia. Nature, 426(6966), 549–552. 10.1038/nature02102 14654838

[ece37408-bib-0065] Kieffer, B. , Arndt, N. , Lapierre, H. , Bastien, F. , Bosch, D. , Pecher, A. , Gezahegn, Y. , Ayalew, D. , Weis, D. , Jerram, D. A. , Keller, F. , & Meugniot, C. (2004). Flood and shiled basalts from Ethiopia: Magmas from the African superswell. Journal of Petrology, 45(4), 793–834. 10.1093/petrology/egg112

[ece37408-bib-0066] Kristjánsson, L. , Hardarson, B. S. , & Audunsson, H. (2003). A detailed palaeomagnetic study of the oldest (≈15 Myr) lava sequences in Northwest Iceland. Geophysical Journal International, 155(3), 991–1005. 10.1111/j.1365-246X.2003.02111.x

[ece37408-bib-0067] Kristjánsson, L. , Pätzold, R. , & Preston, J. (1975). The palaeomagnetism and geology of the Patrekesfjörður‐Arnarfjörður region of Northwest Iceland. Tectonophysics, 25, 201–216.

[ece37408-bib-0068] Lange, S. , Bussmann, R. W. , & Beck, E. (1997). Stand structure and regeneration of the subalpine *Hagenia abyssinica* forests of Mt. Kenya. Botanica Acta, 110(6), 473–480.

[ece37408-bib-0069] Legesse, N. (1995). Indigenous trees of Ethiopia: Biology, use and propagation techniques (p. 386). Addis Ababa University Press.

[ece37408-bib-0070] Li, Y. , Smith, T. , Liu, C. J. , Awasthi, N. , Yang, J. , Wang, Y. F. , & Li, C. S. (2011). Endocarps of *Prunus* (Rosaceae: Prunoideae) from the early Eocene of Wutu, Shandong Province, China. Taxon, 60(2), 555–564.

[ece37408-bib-0071] López‐Sáez, J. A. , & Domínguez‐Rodrigo, M. (2009). Palynology of OGS‐6a and OGS‐7, two new 2.6 Ma archaeological sites from Gona, Afar, Ethiopia: Insights on aspects of late Pliocene habitats and the beginnings of stone‐tool use. Geobios, 42(4), 503–511. 10.1016/j.geobios.2008.12.002

[ece37408-bib-0072] Macgregor, D. S. (2012). The development of the Nile drainage system: Integration of onshore and offshore evidence. Petroleum Geoscience, 18, 417–431. 10.1144/petgeo2011-074

[ece37408-bib-0073] Macgregor, D. (2015). History of the development of the East African Rift System: A series of interpreted maps through time. Journal of African Earth Sciences, 101, 232–252. 10.1016/j.jafrearsci.2014.09.016

[ece37408-bib-0074] Mairal, M. , Sanmartín, I. , Herrero, A. , Pokorny, L. , Vargas, P. , Aldasoro, J. J. , & Alarcón, M. (2017). Geographic barriers and Pleistocene climate change shaped patterns of genetic variation in the Eastern Afromontane biodiversity hotspot. Nature Scientific Reports, 7, 45749. 10.1038/srep45749 PMC5387718 28397796

[ece37408-bib-0075] Matthews, J. V. , Westgate, J. A. , Ovenden, L. , Carter, L. D. , & Fouch, T. (2003). Stratigraphy, fossils, and age of sediments at the upper pit of the Lost Chicken gold mine: New information on the late Pliocene environment of east central Alaska. Quaternary Research, 60(1), 9–18. 10.1016/S0033-5894(03)00087-5

[ece37408-bib-0076] McDougall, I. , Kristjansson, L. , & Saemundsson, K. (1984). Magnetostratigraphy and eochronology of northwest Iceland. Journal of Geophysical Research, 89(B8), 7029–7060. 10.1029/JB089iB08p07029

[ece37408-bib-0077] Measey, G. J. , & Tolley, K. A. (2011). Sequential fragmentation of Pleistocene forests in an east Africa biodiversity hotspot: Chameleons as a model to track forest history. PLoS One, 6(10), e26606. 10.1371/journal.pone.0026606 22053198 PMC3203880

[ece37408-bib-0078] Merckx, V. S. F. T. , Hendriks, K. P. , Beentjes, K. K. , Mennes, C. B. , Becking, L. E. , Peijnenburg, K. T. C. A. , Afendy, A. , Arumugam, N. , de Boer, H. , Biun, A. , Buang, M. M. , Chen, P.‐P. , Chung, A. Y. C. , Dow, R. , Feijen, F. A. A. , Feijen, H. , Soest, C.‐V. , Geml, J. , Geurts, R. , … Schilthuizen, M. (2015). Evolution of endemism on a young tropical mountain. Nature, 524(7565), 347–350. 10.1038/nature14949 26266979

[ece37408-bib-0079] Miller, M. A. , Pfeiffer, W. , & Schwartz, T. (2010). Creating the CIPRES Science Gateway for inference of large phylogenetic trees. In: Proceedings of the Gateway Computing Environments Workshop (GCE), New Orleans, LA, 14 November 2010. 1–8. 10.1109/GCE.2010.5676129

[ece37408-bib-0080] Mittermeier, R. A. , Gil, P. R. , Hoffmann, M. , Pilgrim, J. , Brooks, T. , Mittermeier, C. G. , Lamoreux, J. , & Da Fonseca, G. A. B. (2004). Hotspots Revisited. CEMEX Books on Nature Series, Patricia Robles Gil, Producer. p. 200.

[ece37408-bib-0081] Moorbath, S. , Sigurdsson, H. , & Goodwin, R. (1968). K‐Ar ages of the oldest exposed rocks in Iceland. Earth and Planetary Science Letters, 4(3), 197–205. 10.1016/0012-821X(68)90035-6

[ece37408-bib-0082] Negash, L. (2010). A Selection of Ethiopia's Indigenous Trees: Biology, Uses, and Propagation Techniques (p. 386). Addis Ababa University Press.

[ece37408-bib-0083] Olago, D. O. , Street‐Perrott, F. A. , Perrott, R. A. , Ivanovich, M. , & Harkness, D. D. (1999). Late Quaternary glacial‐interglacial cycle of climatic and environmental change on Mount Kenya. Kenya. Journal of African Earth Sciences, 29(3), 593–618. 10.1016/S0899-5362(99)00117-7

[ece37408-bib-0084] Orwa, C. , Mutua, A. , Kindt, R. , Jamnadass, R. , & Anthony, S. (2009). Agroforestree Database: a tree reference and selection guide version 4.0 [online]. Available from http://apps.worldagroforestry.org/treedb2/. Accessed November 2019.

[ece37408-bib-0085] Pan, A. D. , Currano, E. D. , Jacobs, B. F. , Feseha, M. , Tabor, N. , & Herendeen, P. S. (2012). Fossil *Newtonia* (Fabaceae) seeds from the early Miocene (22–21 Ma) Mush Valley in Ethiopia. International Journal of Plant Sciences, 173(3), 290–296. 10.1086/663967

[ece37408-bib-0086] Pan, A. D. , Jacobs, B. F. , & Currano, E. D. (2014). Dioscoreaceae fossils from the Late Oligocene and early Miocene of Ethiopia. Botanical Journal of the Linnean Society, 175(1), 17–28. 10.1111/boj.12150

[ece37408-bib-0087] Pérez De Paz, J. (2004). Rosaceae‐Sanguisorbeae de Macaronesia: Géneros Marcetella, Bencomia, y Dendriopterium. Palinología, Biogeografía. Systemas Sexuales Y Filogenia. Botánica Macaronesica, 25, 95–126.

[ece37408-bib-0088] Piel, W. H. , Chan, L. , Dominus, M. J. , Ruan, J. , Vos, R. A. , & Tannen, V. (2009). TreeBASE v. 2: A Database of Phylogenetic Knowledge. e‐BioSphere.

[ece37408-bib-0089] Pik, R. , Marty, B. , Carignan, J. , & Lave, J. (2003). Stability of the Upper Nile drainage network (Ethiopia)deduced from (U‐Th)He thermochronometry: Implications for uplift and erosion of the Afar plume dome. Earth and Planetary Science Letters, 215(1–2), 73–88. 10.1016/S0012-821X(03)00457-6

[ece37408-bib-0090] Potter, D. , Eriksson, T. , Evans, R. C. , Oh, S. , Smedmark, J. E. E. , Morgan, D. R. , Kerr, M. , Robertson, K. R. , Arsenault, M. , Dickinson, T. A. , & Campbell, C. S. (2007). Phylogeny and classification of Rosaceae. Plant Systematics and Evolution, 266(1‐2), 5–43. 10.1007/s00606-007-0539-9

[ece37408-bib-0091] POWO (2019). Plants of the World Online. http://www.plantsoftheworldonline.org. Accessed July 2019.

[ece37408-bib-0092] Prothero, D. R. , & Sanchez, F. (2004). Magnetic stratigraphy of the upper Eocene Florissant formation, Teller county, Colorado. In: Lucas SG, Zeigler KE, Kondrashov PE, eds. Paleogene Mammals. New Mexico Museum of Natural History and Science Bulletin, 26, 129–135.

[ece37408-bib-0093] Punt, W. , Bos, J. A. A. , & Hoen, PP (1991). The Northwest European Pollen Flora, 45. Oleaceae. Review of Palaeobotany and Palynology, 69(1–3), 23–47. 10.1016/0034-6667(91)90065-B

[ece37408-bib-0094] Punt, W. , Hoen, P. P. , Blackmore, S. , Nilsson, S. , & Le Thomas, A. (2007). Glossary of pollen and spore terminology. Review of Palaeobotany and Palynology, 143(1–2), 1–81. 10.1016/j.revpalbo.2006.06.008

[ece37408-bib-0095] Rahbek, C. , Borregaard, M. K. , Antonelli, A. , Colwell, R. K. , Holt, B. G. , Nogues‐Bravo, D. , Rasmussen, C. M. , Richardson, K. , Rosing, M. T. , Whittaker, R. J. , & Fjeldså, J. (2019). Building mountain biodiversity: Geological and evolutionary processes. Science, 365(6458), 1114–1119. 10.1126/science.aax0151 31515384

[ece37408-bib-0096] Read, P. B. (2000). Geology and industrial minerals of the Tertiary basins, south‐central British Columbia. Ministry of Energy and Mines, p. 110.

[ece37408-bib-0097] Ronquist, F. , Teslenko, M. , Van Der Mark, P. , Ayres, D. L. , Darling, A. , Höhna, S. , Larget, B. , Liu, L. , Suchard, M. A. , & Huelsenbeck, J. P. (2012). MrBayes 3.2: Efficient Bayesian phylogenetic inference and model choice across a large model space. Systematic Biology, 61(3), 539–542. 10.1093/sysbio/sys029 22357727 PMC3329765

[ece37408-bib-0098] Schüler, L. , Hemp, A. , & Behling, H. (2014). Relationship between vegetation and modern pollen‐rain along and elevational gradient on Kilimanjaro, Tanzania. Holocene, 24(6), 702–713. 10.1177/0959683614526939

[ece37408-bib-0099] Schüler, L. , Hemp, A. , Zech, W. , & Behling, H. (2012). Vegetation, climate and fire‐dynamics in East Africa inferred from the Maundi crater pollen record from Mt Kilimanjaro during the last glacial‐interglacial cycle. Quaternary Science Reviews, 39, 1–13. 10.1016/j.quascirev.2012.02.003

[ece37408-bib-0100] GBIF Secretariat (2019). *Hagenia abyssinica* J.F.Gmel. In: GBIF Backbone Taxonomy. Checklist dataset. 10.15468/39omei [accessed via GBIF.org on 2021‐02‐01]

[ece37408-bib-0101] Sembroni, A. , Molin, P. , Pazzaglia, F. J. , Faccenna, C. , & Bekele, A. (2016). Evolution of continental‐scale drainage in response to mantle dynamics and surface processes: An example from the Ethiopian Highlands. Geomorphology, 261, 12–29. 10.1016/j.geomorph.2016.02.022

[ece37408-bib-0102] Simion, T. (2018). Kosso (Hagenia abyssinica (Bruce) J.F.Gmel.) Genetic Resource. Agricultural Research & Technology: Open Access Journal, 16(3), 10.19080/ARTOAJ.2018.16.555987

[ece37408-bib-0103] Smith, M. E. , Singer, B. , & Carroll, A. (2003). 40Ar/39Ar geochronology of the Eocene Green River Formation, Wyoming. GSA Bulletin, 115(5), 549–565. 10.1130/0016-7606(2003)115<0549:AGOTEG>2.0.CO;2

[ece37408-bib-0104] Smith, S. A. (2019). SortaDate, Github repository. https://github.com/FePhyFoFum/SortaDate

[ece37408-bib-0105] Smith, S. A. , Brown, J. W. , & Walker, J. F. (2018). So many genes, so little time: A practical approach to divergence‐time estimation in the genomic era. PLoS One, 13(5), e0197433. 10.1371/journal.pone.0197433 29772020 PMC5957400

[ece37408-bib-0106] Spehn, E. M. , Rudmann‐Maurer, K. , & Körner, C. (2011). Mountain biodiversity. Plant Ecology & Diversity, 4(4), 301–302. 10.1080/17550874.2012.698660

[ece37408-bib-0107] Stamatakis, A. (2014). RAxML version 8: A tool for phylogenetic analysis and post‐analysis of large phylogenies. Bioinformatics, 30(9), 1312–1313. 10.1093/bioinformatics/btu033 24451623 PMC3998144

[ece37408-bib-0108] Tadesse, S. , Milesi, J.‐P. , & Deschamps, Y. (2003). Geology and mineral potential of Ethiopia: A note on geology and mineral map of Ethiopia. Journal or African Earth Sciences, 36(4), 273–313. 10.1016/S0899-5362(03)00048-4

[ece37408-bib-0109] Taylor, D. (1990). Late quaternary pollen records from two Ugandan mires: Evidence for environmental changes in the Rukiga highlands of southwest Uganda. Palaeogeography, Palaeoclimatology, Palaeoecology, 80(3–4), 283–300. 10.1016/0031-0182(90)90138-W

[ece37408-bib-0110] Tesfamichael, T. , Jacobs, B. F. , Tabor, N. , Michel, L. , Currano, E. , Feseha, M. , Barclay, R. , Kappelmann, J. , & Schmitz, M. (2017). Settling the issue of “decoupling” between atmospheric carbon dioxide and global temperature: [CO_2_]_atm_ reconstructions across the warming Paleogene‐Neogene divide. Geology, 45(11), 999–1002. 10.1130/G39048.1

[ece37408-bib-0111] British Geological Survey . (2020). The BGS Lexicon of Named Rock Units [online]. Keyworth, Nottingham. Available from https://www.bgs.ac.uk/technologies/the‐bgs‐lexicon‐of‐named‐rock‐units/. https://www.bgs.ac.uk/lexicon/lexicon.cfm?pub=BOSS. Accessed January 2020.

[ece37408-bib-0112] Ulrich, S. , & Grímsson, F. (2020). The single‐grain method: Adding TEM to the equation. Grana, 59(1), 44–57. 10.1080/00173134.2019.1666915 32256293 PMC7077360

[ece37408-bib-0113] Ulrich, S. , & Tweraser, E. (2016). Salvia verticillata . In: PalDat – A palynological database. https://www.paldat.org/pub/Salvia_verticillata/300513. Accessed December 09, 2019.

[ece37408-bib-0114] Umer, M. , Lamb, H. F. , Bonnefille, R. , Lezine, A. M. , Tiercelin, J. J. , Gibert, E. , Cazet, J. P. , & Watrin, J. (2007). Late pleistocene and holocene vegetation history of the Bale Mountains, Ethiopia. Quaternary Science Reviews, 26(17–18), 2229–2246. 10.1016/j.quascirev.2007.05.004

[ece37408-bib-0115] UTPD (2014). Useful Tropical Plants Database. http://www.tropical.theferns.info. Accessed June 2019.

[ece37408-bib-0116] Vallejo‐Marín, M. (2019). Buzz pollination: Studying bee vibrations on flowers. New Phytologist, 224(3), 1068–1074. 10.1111/nph.15666 30585638

[ece37408-bib-0117] Vergel, M. M. , & Durango de Cabrera, J. (1988). Palinología de la Formación Cullen (Terciario) de las inmediaciones de Cañadón Beta, Tierra del Fuego, República Argentina. In: Proceedings of the 5th Congreso Geológico Chileno, Santiago, Chile, 8‐12 August, 1988. C227–C245

[ece37408-bib-0118] Weber, M. , & Ulrich, S. (2010). The endexine: A frequently over‐looked pollen wall layer and a simple method for detection. Grana, 49(2), 83–90. 10.1080/00173131003743949

[ece37408-bib-0119] White, F. (1979). The Guineo‐Congolian region and its relationships to other phytochoria. Bulletin Du Jardin Botanique National De Belgique/Bulletin Van De Nationale Plantentuin Van België, 49(1/2), 11–55.

[ece37408-bib-0120] White, F. (1983). The Vegetation of Africa (p. 354). Unesco, United Nations.

[ece37408-bib-0121] White, F. (1993). The AETFAT chorological classification of Africa: History, methods and application. Bulletin Du Jardin Botanique National De Belgique/Bulletin Van De Nationale Plantentuin Van België, 62(1/4), 225–281. 10.2307/3668279

[ece37408-bib-0122] Willmer, P. (2011). Pollination and floral ecology (p. 832). Princeton University Press.

[ece37408-bib-0123] Wolde, T. , Bizuayehu, B. , Hailemariam, T. , & Tiruha, K. (2016a). Phytochemical analysis and antimicrobial activity of *Hagenia abyssinica*. *Indian* . Journal of Pharmacy and Pharmacology, 3(3), 127–134. 10.5958/2393-9087.2016.00028.5

[ece37408-bib-0124] Wolde, T. , Bizuayehu, B. , Hailemariam, T. , & Tiruha, K. (2016b). Phytochemical analysis and antimicrobial activity of *Hagenia abyssinica* . International Journal of Advanced Research in Chemical Science, 3(8), 15–23. 10.20431/2349-0403.0308003

[ece37408-bib-0125] Wolfe, J. A. , Gregory‐Wodzicki, K. M. , Molnar, P. , & Mustoe, G. (2003). Rapid uplift and then collapse in the Eocene of the Okanagan? Evidence from paleobotany. In: *Geological Association of Canada*–Mineralogical Association of Canada–Society Economic Geologists, Joint Annual Meeting, Vancouver, Abstracts, 28: 533.

[ece37408-bib-0126] Xiang, Y. , Huang, C. H. , Hu, Y. , Wen, J. , Li, S. , Yi, T. , Chen, H. , Xiang, J. , & Ma, H. (2017). Evolution of Rosaceae fruit types based on nuclear phylogeny in the context of geological times and genome duplication. Molecular Biology and Evolution, 34(2), 262–281. 10.1093/molbev/msw242 27856652 PMC5400374

[ece37408-bib-0127] Yemane, K. , Bonnefille, R. , & Faure, H. (1985). Palaeoclimatic and tectonic implications of Neogene microflora from the Northwestern Ethiopian highlands. Nature, 318, 653–656. 10.1038/318653a0

[ece37408-bib-0128] Yemane, K. , Robert, C. , & Bonnefille, R. (1987). Pollen and clay mineral assemblages of a late Miocene lacustrine sequence from the northwestern Ethiopian highlands. Palaeogeography, Palaeoclimatology, Palaeoecology, 60, 123–141. 10.1016/0031-0182(87)90028-9

[ece37408-bib-0129] Yeo, P. F. (1993). Secondary pollen presentation, form, function and evolution. Plant Systematics and Evolution Supplementum, 6, 1–268. 10.1007/978-3-7091-6670-3

[ece37408-bib-0130] Young, N. E. , Romme, W. H. , Evangelista, P. H. , Mengistu, T. , & Worede, A. (2017). Variation in population structure and dynamics of montane forest tree species in Ethiopia guide priorities for conservation and research. Biotropica, 49(3), 309–317. 10.1111/btp.12420

[ece37408-bib-0131] Zachos, J. C. , Dickens, G. R. , & Zeebe, R. E. (2008). An early Cenozoic perspective on greenhouse warming and carbon‐cycle dynamics. Nature, 451, 279–283. 10.1038/nature06588 18202643

[ece37408-bib-0132] Zamaloa, M. C. (1996). Asociación de zigospóras de Zygnemataceae (Chlorophyta) en el Terciario medio de Tierra del Fuego, Argentina. Ameghiniana, 33, 179–184.

[ece37408-bib-0133] Zamaloa, M. C. (1999). Estudio palinológico de la Formación Cullen (Terciario superior), Tierra del Fuego, Argentina. Facultad de Ciencias Exactas y Naturales. Universidad de Buenos Aires. http://digital.bl.fcen.uba.ar/Download/Tesis/Tesis_3187_Zamaloa.pdf

[ece37408-bib-0134] Zamaloa, M. C. (2000). Palinoflora y ambiente en el Terciario del nordeste de Tierra del Fuego, Argentina. Revista Del Museo Argentino De Ciencias Naturales, 2(1), 43–51. 10.22179/REVMACN.2.123

[ece37408-bib-0135] Zamaloa, M. C. (2004). Miocene algae and spores from Tierra del Fuego. Argentina. Alcheringa, 28(1), 205–227. 10.1080/03115510408619282

[ece37408-bib-0136] Zamaloa, M. C. , & Romero, E. J. (1990). Some spores and pollen from the Cullen formation (upper Eocene to middle Oligocene), Tierra del Fuego, Argentina. Palynology, 14(1), 123–133. 10.1080/01916122.1990.9989376

[ece37408-bib-0137] Zamaloa, M. C. , & Romero, E. J. (2005). Neogene palynology of Tierra del Fuego, Argentina: Conifers. Alcheringa, 29(1), 113–121. 10.1080/03115510508619563

[ece37408-bib-0138] Zetter, R. (1989). Methodik und Bedeutung einer routinemäßig kombinierten lichtmikroskopischen und rasterelektonenmikroskopischen Untersuchung fossiler Mikrofloren. Courier Forschungsinstitut Senckenberg, 109, 41–50.

[ece37408-bib-0139] Zetter, R. , Hofmann, C.‐C. , Draxler, I. , Durango de Cabrera, J. , Vergel, M. , & Vervoorst, F. (1999). A rich middle Eocene microflora at Arroyo de los Mineros, near Cañadón beta, NE Tierra del Fuego Province, Argentina. Abhandlungen Der Geologischen Bundesanstalt, 56, 439–460.

[ece37408-bib-0140] Zhang, S. D. , Jin, J. J. , Chen, S. Y. , Chase, M. W. , Soltis, D. E. , Li, H. T. , Yang, J. B. , Li, D. Z. , & Yi, T. S. (2017). Diversification of Rosaceae since the Late Cretaceous based on plastid phylogenomics. New Phytologist, 214(3), 1355–1367. 10.1111/nph.14461 28186635

