# Supplementary figures and images for: Hagenia from the early Miocene of Ethiopia: Evidence for possible niche evolution?
Source: Ecol Evol. 2021 Mar 23;11(10):5164–86. doi: 10.1002/ece3.7408 (PMC8131786; doi:10.1002/ece3.7408)

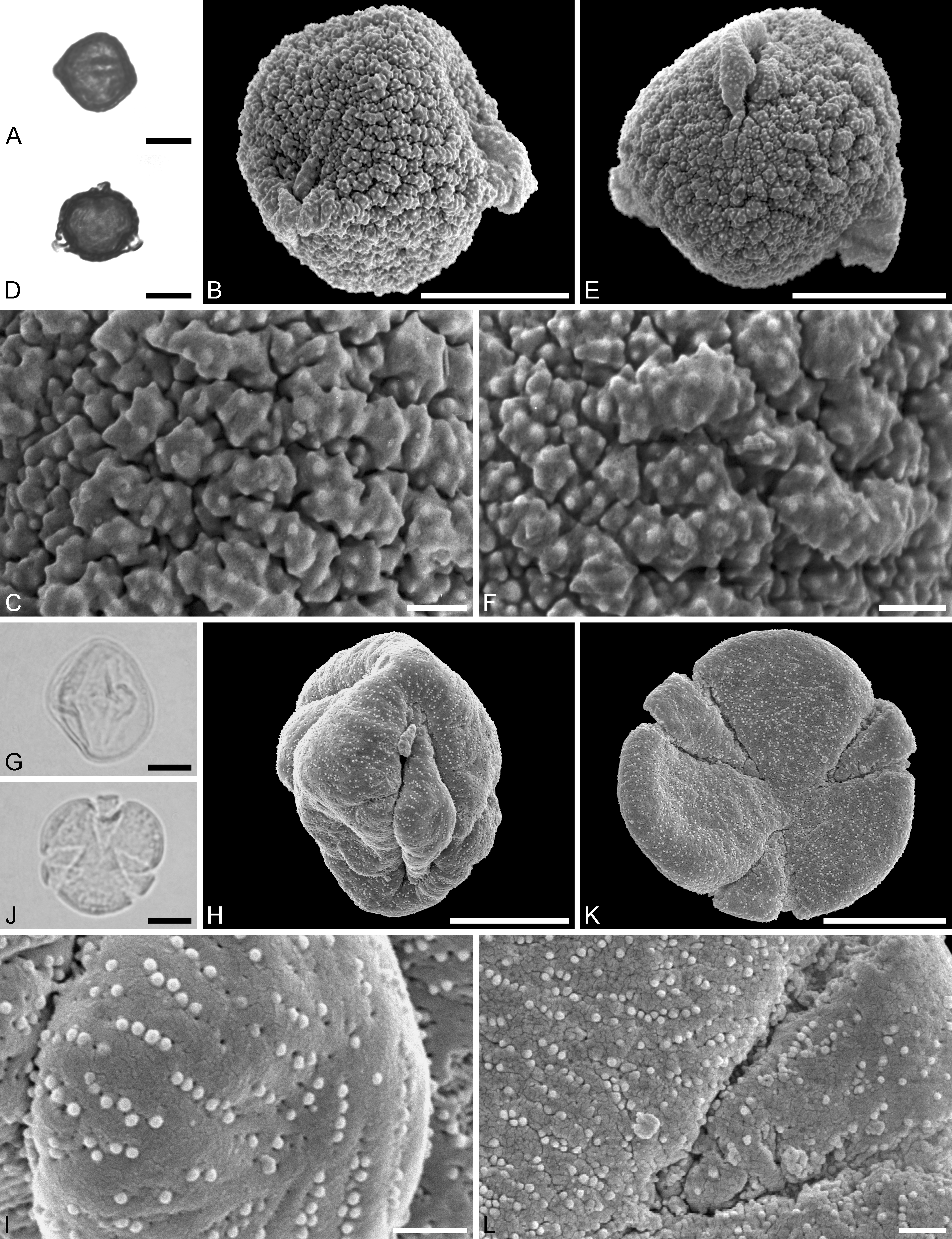

Supplement: Supplementary file 1 — Figure S1 [file ECE3-11-5164-s001.tif]
