# Supplementary material for: Hagenia from the early Miocene of Ethiopia: Evidence for possible niche evolution?
Source: Ecol Evol. 2021 Mar 23;11(10):5164–86. doi: 10.1002/ece3.7408 (PMC8131786; doi:10.1002/ece3.7408)
Supplement: Supplementary file 2 — Supplementary Material [file ECE3-11-5164-s002.doc]

**Text to Appendix Figure.**

Light microscopy (**A, D, G, J**) and scanning electron microscopy (**B, C, E, F, H, I, K, L**) micrographs of fossil *Acaena* vel *Polylepis* pollen (Arroya de los Mineros; same grain: **A–C**; same grain: **D–F**) and *Sanguisorba* pollen (Botn; same grain: **G–I**; same grain: **J–L**). **A.** Equatorial view. **B.** Equatorial view view. **C.** Close-up of interapertural area. **D.** Polar view. **E.** Oblique polar view. **F.** Close-up of polar area. **G.** Equatorial view. **H.** Equatorial view. **I.** Close-up of aperture, showing operculum. **J.** Polar view. **K.** Polar view. **L.** Close-up of area surrounding operculum in polar region. Scale bars – 10 µm (A, B, D, E, G, H, J, K), 1 µm (C, F, I, L).
